# Land use gradients drive spatial variation in Lassa fever host communities in the Eastern Province of Sierra Leone

**DOI:** 10.1111/1365-2656.70187

**Published:** 2025-11-21

**Authors:** David Simons, Rory Gibb, Umaru Bangura, Dianah Sondufu, Joyce Lamin, James Koninga, Momoh Foday, Mike Dawson, Joseph Lahai, Rashid Ansumana, Elisabeth Fichet‐Calvet, Deborah Watson‐Jones, Richard Kock, Kate E. Jones

**Affiliations:** ^1^ Centre for Emerging, Endemic and Exotic Diseases, The Royal Veterinary College London UK; ^2^ Department of Genetics, Evolution and Environment, Centre for Biodiversity and Environment Research University College London London UK; ^3^ Clinical Research Department London School of Hygiene and Tropical Medicine London UK; ^4^ People & Nature Lab, UCL East, Department of Genetics, Evolution and Environment University College London London UK; ^5^ Bernard‐Nocht Institute for Tropical Medicine, WHO Collaborating Centre for Arbovirus and Hemorrhagic Fever Reference and Research Hamburg Germany; ^6^ Njala University Bo Sierra Leone; ^7^ Kenema Government Hospital Kenema Sierra Leone; ^8^ Mwanza Intervention Trials Unit, National Institute for Medical Research Mwanza Tanzania

**Keywords:** biodiversity and zoonotic risk, disease ecology, infectious diseases, Lassa fever, rodent‐associated zoonoses, small‐mammal community dynamics

## Abstract

1. The natal multimammate mouse (*Mastomys natalensis*) is the primary reservoir host of *Mammarenavirus lassaense* (LASV), a zoonotic pathogen causing Lassa fever and endemic to West Africa. The occurrence and abundance of this species is regulated by the human environment and biotic interactions with other small‐mammal species, but these ecological drivers remain poorly understood in the regions where Lassa fever outbreaks are observed.
2. We developed a Bayesian multi‐species occupancy model incorporating incomplete detection to assess habitat use from data obtained as part of a multi‐year small‐mammal trapping study (43,226 trap nights across four village sites in Sierra Leone, 2020–2023). We investigated the effects of land use gradients and small‐mammal community dynamics on the spatial distribution of *M. natalensis*.
3. *Mastomys natalensis* occupancy increased along a gradient from forest to agriculture to village habitats but was reduced in the peri‐urban site compared to rural settings. Invasive rodent species influenced this pattern, with *Mus musculus* presence associated with reduced *M. natalensis* occupancy in the peri‐urban site. We did not observe a similar effect when considering the co‐occurrence of invasive *Rattus rattus* with *M. natalensis* in rural settings.
4. These findings suggest that land use and species interactions drive spatial heterogeneity in *M. natalensis* populations, potentially explaining reduced Lassa fever incidence in urban areas. The results highlight the importance of considering community dynamics when predicting the risk of outbreaks of endemic zoonoses and the need to widen the context of studies of LASV transmission beyond the primary reservoir host species.
5. To better assess public health risk and improve allocation of limited resources, we recommend more precise characterisation of small‐mammal communities in LASV endemic regions, particularly in areas undergoing rapid land use change which may alter community‐level small‐mammal biodiversity.

## Graphical abstract

As West Africa urbanises, the risk of Lassa fever may paradoxically decrease. We found the invasive house mouse, a dominant urban species, outcompetes and displaces the primary Lassa virus host. Considering these species interactions is critical for accurately predicting future zoonotic disease patterns.

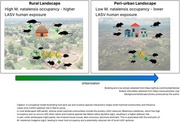

## 1 INTRODUCTION

Global biodiversity is in decline, with biodiversity loss directly influencing zoonotic disease risk (Halliday et al., 2020; IPBES, 2020; Mantyka‐Pringle et al., 2015; Sala et al., 2000). Land use change, particularly the conversion of natural habitats into agricultural or urban landscapes, is a key driver of biodiversity loss, reducing mammalian species diversity across several dimensions (Newbold et al., 2015). These include taxonomic diversity (the number and relative abundance of taxa), functional diversity (the range of roles organisms play within an ecosystem) and interaction diversity (the biotic interactions among species) (Glidden et al., 2021; Naeem et al., 2012). These declines in biodiversity, particularly in rodent‐associated disease systems, may exacerbate zoonotic disease risks by promoting the proliferation of generalist, synanthropic rodents that thrive in human‐modified landscapes, where they host zoonotic pathogens (Ecke et al., 2022; Gibb et al., 2020; Young et al., 2014). This shift may therefore not only increase the prevalence of zoonotic pathogens but also human exposure in complex socio‐ecological systems (Gibb et al., 2025).

The role of host community diversity in mediating zoonotic outbreaks is nuanced, with changes in species composition and pathogen prevalence often interacting with anthropogenic stressors to create context‐specific outcomes (Carlson et al., 2025; Gibb et al., 2020; Keesing & Ostfeld, 2021). A deeper mechanistic understanding of these processes, including community structure, biotic interactions and responses to anthropogenic land use change, is critical for leveraging biodiversity knowledge to predict and mitigate zoonotic risks (Carlson et al., 2025; Glidden et al., 2021; Salkeld et al., 2013). These approaches have only been taken for a few rodent‐associated zoonoses (Keesing & Ostfeld, 2024).

Rodents are an important mammalian host taxa for zoonotic diseases (Han et al., 2015; Mendoza et al., 2019). Rodent‐associated zoonoses, such as Lyme disease caused by *Borrelia burgdorferi* sensu lato, have been shown to involve a complex interplay of community structure, biotic interactions, and the effect of land use change (Ostfeld & Holt, 2004). For instance, land use change can increase reservoir abundance and subsequently zoonotic risk, though responses vary across systems and settings, highlighting the idosyncratic or system specific nature of these dynamics (Mendoza et al., 2019; Pei et al., 2024; Young et al., 2017).

Lassa fever, caused by *Mammarenavirus lassaense* (LASV), is an important rodent‐associated zoonosis endemic to West Africa. It is reported from Nigeria, Guinea, Sierra Leone, Liberia, Mali, Benin, Ghana and Togo and causes an estimated 900,000 annual infections with substantial morbidity and mortality (Basinski et al., 2021; World Health Organisation, 2022). Risk of infection in human populations is spatially heterogeneous with patchy distributions of reported cases across the endemic region (Agbonlahor et al., 2021; Gibb et al., 2017; Grant et al., 2023). While epidemiological factors such as acquired immunity and asymptomatic infections may contribute, reservoir host ecology and distribution are likely critical drivers of this variation, though heterogeneous reporting effort may also be a contributing factor (McCormick et al., 1987; Redding et al., 2021; Simons, 2022).

*Mastomys natalensis*, considered the main reservoir host of LASV, is a synanthropic rodent species found throughout sub‐Saharan Africa (IUCN, 2016; Olayemi, Obadare, et al., 2016). It is abundant in and around areas of human‐dominated landscapes where it is considered a pest species (Leirs et al., 1993). Population dynamics in *M. natalensis* are influenced by resource availability and seasonal rainfall pulses, which drive fluctuations in abundance that coincide with increased human Lassa fever outbreaks (Leirs et al., 1997; Leirs, Verhagen, et al., 1996; Redding et al., 2021). However, most intensive ecological studies of *M. natalensis* have been conducted outside the Lassa fever endemic region, in regions with differing agro‐ecologies, potentially limiting their applicability to West Africa (Leirs, Verhagen, et al., 1996). The effects of land use change on rodent communities in the endemic region have only been explored across a limited geographic area, with most studies focusing solely on *M. natalensis* (Arruda et al., 2021; Fichet‐Calvet et al., 2005, 2014; Fichet‐Calvet et al., 2009; Olayemi et al., 2018). The limited number of studies that do consider the wider rodent community typically focus on abundance (measured through summary proxies such as trap success) rather than explicitly investigating community dynamics (Eskew et al., 2024; Fichet‐Calvet et al., 2009).

There is a pressing need for systematic small‐mammal community studies in West Africa to ensure that insights from other rodent‐associated zoonoses can be generalized to Lassa fever. Such studies can help disentangle the respective roles of abiotic factors, such as land use type, and biotic interactions within small‐mammal communities in shaping *M. natalensis* distributions and pathogen exposure risks. *Mastomys natalensis* occurs within species‐rich settings in West Africa; however, the biotic interactions between this species and other native and invasive rodents within these communities, and how these interactions may regulate *M. natalensis* occurrence are not well described (Cuypers et al., 2017; Fichet‐Calvet et al., 2009; Garba et al., 2014; Hima et al., 2019). Past studies have indicated that competitive interactions with *Rattus rattus* and *Mus musculus* can alter native rodent communities, and might therefore reshape *M. natalensis* local distributions with subsequent effects on LASV transmission and Lassa fever hazard (Dalecky et al., 2015; Eskew et al., 2024; Lippens et al., 2017). Sierra Leone is associated with frequent outbreaks of Lassa fever in human populations, with evidence of outbreaks beyond the traditionally accepted endemic region, but the influence of community dynamics on these patterns remains poorly understood (Bangura et al., 2021; Bonner et al., 2007; Bonwitt et al., 2017; Eskew et al., 2024; Grant et al., 2023; Keenlyside et al., 1983; McCormick et al., 1987; Monath et al., 1974).

Given these gaps in understanding, we conducted repeated, systematic small‐mammal trapping along a land use gradient in Eastern Sierra Leone to explore how small‐mammal community structure influences the distribution of *M. natalensis*. We hypothesised that (1) small‐mammal community diversity varies with land use, with lower diversity in more anthropogenic habitats; (2) *M. natalensis* occupancy is positively associated with anthropogenic land use but negatively influenced by competition with other sympatric species; and (3) species interactions within small‐mammal communities regulate the local distribution of *M. natalensis*, thereby shaping LASV outbreak risk. Sierra Leone, a hotspot for Lassa fever, provides a unique opportunity to explore these dynamics in a region where rodent ecology studies have largely focused on *M. natalensis*, often neglecting the broader small‐mammal community. We expect these analyses to further understanding of small‐mammal community structures that may explain observed patterns of LASV outbreaks within this context and the wider endemic region.

## 2 METHODS

### 2.1 Small‐mammal sampling

**FIGURE 1:**
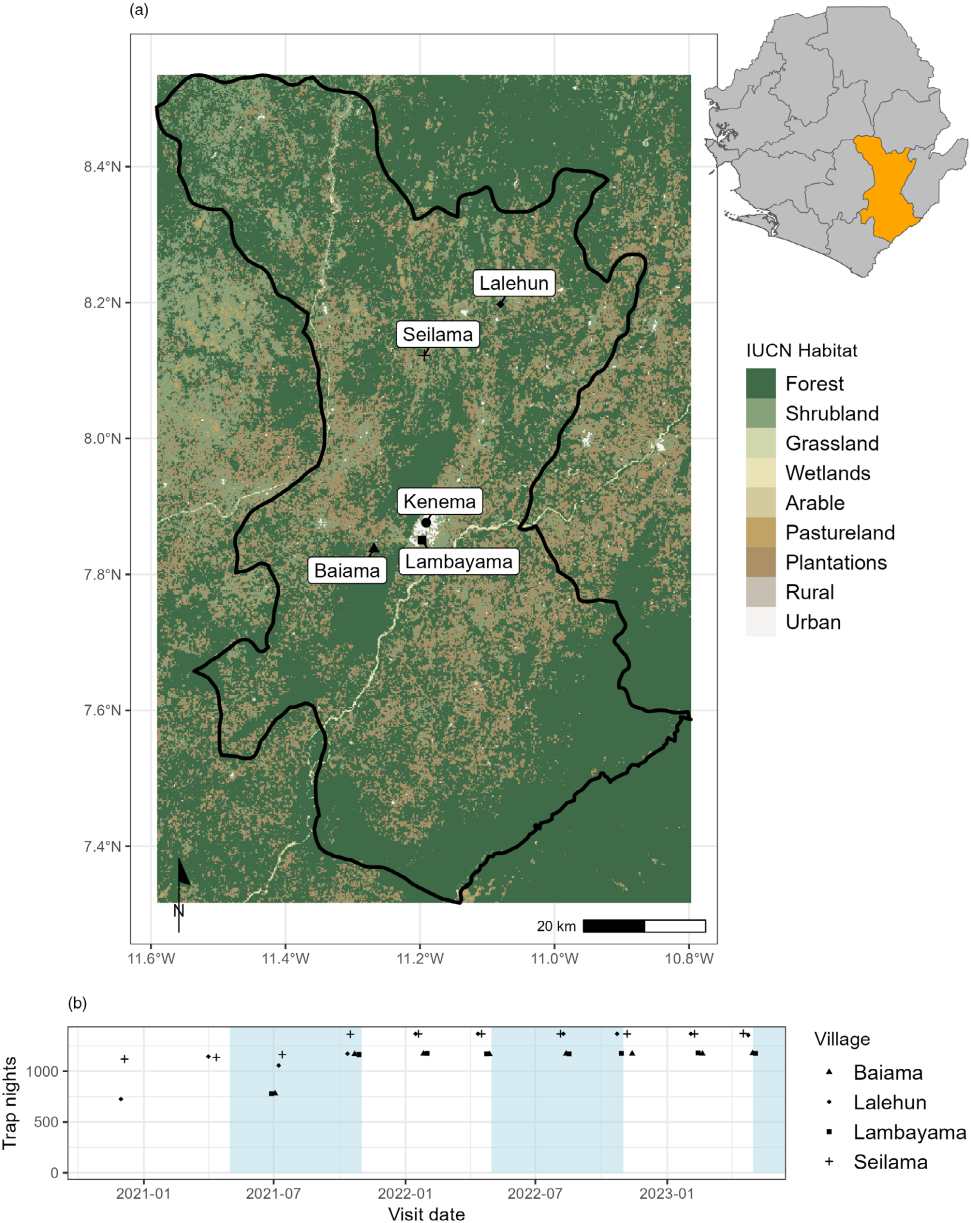
Village site locations and dates of rodent trapping in Sierra Leone. (a) Location of village study sites (cross—Seilama, diamond—Lalehun, triangle—Baiama, square—Lambatama), in the Eastern Province of Sierra Leone. Kenema, the largest city of the province is shown with a circle. The inset map shows the location of Kenema District in Sierra Leone. (b) Number of trap nights obtained from each study village, blue shaded regions represent the rainy season in Sierra Leone. The background satellite image of Eastern Province, Sierra Leone obtained from Google Maps copyright TerraMetrics 2023.

We conducted small‐mammal trapping surveys from October 2020 to April 2023 at four village study sites (Baiama, Lalehun, Lambayama and Seilama) located in the Lassa fever endemic zone of Sierra Leone's Eastern Province (Figure 1a). Site selection was informed by discussions with the Kenema Government Hospital Lassa Fever team and guided by remote imaging data to ensure representation of prior disease outbreak areas. Trapping grids were established along a gradient of anthropogenic disturbance, encompassing forest, agricultural land (both fallow and active) and village areas (inside and outside permanent structures). Each village study site was assigned one forest grid, three to four agricultural grids and two village grids, except for Lambayama, which lacked forested land (Figure S1A–D). Trapping survey sessions within each village occurred four times annually with two trapping surveys in each of the rainy and dry seasons (May to November and December to April, respectively). This produced a total of 10 trapping sessions at Lalehun and Seilama, and 8 trapping sessions at Baiama and Lambayama over the study period due to logistical constraints (Figure 1b).

To explore the influence of human population density on small‐mammal communities, we classified village study sites as either rural or peri‐urban. This classification was based on population density data from WorldPop for 2020 at a 100 m resolution (WorldPop, 2025). We adopted the classifications proposed by Dijkstra et al for country specific density thresholds defining urban centres as those with a density in excess of 1000 individuals/km^2^ and a contiguous population of at least 50,000 (Dijkstra et al., 2021). Under this definition, Lambayama (5227 individuals/km^2^) was classified as peri‐urban (an urban cluster in their manuscript), while Baiama (131 individuals/km^2^), Lalehun (159 individuals/km^2^) and Seilama (48 individuals/km^2^) were classified as rural.

We selected village study sites and trapping grids to be representative of land use in the Eastern Province of Sierra Leone, based on accessibility and acceptability of the study protocol to communities (Supporting Information S1). Trapping grids consisted of 49 Sherman traps (7.62 × 8.89 × 22.86 cm; H.B. Sherman Traps, Tallahassee, USA), arranged in a 7 × 7 layout with 7‐metre spacing between traps, adjusted to the local terrain (median grid area = 1672 m^2^). For grids within permanent structures, the grid arrangement was modified: individual buildings were semi‐randomly selected using a projected village grid, and four traps were deployed per structure. The location of each individual trap was geolocated. Traps were baited with a locally produced mixture of oats, palm oil and dried fish. Each morning, traps were checked and closed for the day prior to re‐baiting during the evening. Each trapping survey session consisted of four consecutive trap nights (TN) at each trapping grid.

For downstream analysis, we used geospatial software to assign individual trap locations to a standardized grid cell system based on their physical coordinates (Figure S2). First, a convex hull encompassing all trapping sessions for a grid was generated. A regular grid composed of 49 m^2^ cells was overlaid on this polygon, and traps were assigned to cells. This process resulted in 2068 unique grid cells across all sites. Grid cells were included in the analysis even if they were only sampled during a single trapping session, as the occupancy model framework can accommodate sites with varying numbers of survey replicates. The four consecutive trap‐nights from each trap within a grid cell during a single survey were pooled as a single replicate for the statistical analysis. Geospatial processing was performed using the sf package in R (version 4.1.2) (Pebesma, 2018; R Core Team, 2021).

All small mammals were handled by trained researchers wearing appropriate personal protective equipment. Animals were sedated using halothane and euthanized according to established protocols (Fichet‐Calvet, 2014). Morphological measurements and samples of blood and tissue were collected. The study was approved by the Clinical Research Ethical Review Board and Animal Welfare Ethical Review Board of the Royal Veterinary College, UK (URN: 2019 1949‐3), and the ethical review board of Njala University, Sierra Leone, and adhered to national and institutional ethical guidelines. Sex was determined based on external and internal genitalia. Age classification was informed by reproductive status and lens weight from dried eye samples. Photographs of the dorsal and ventral aspects of each rodent were taken for morphological documentation. All carcasses were incinerated to mitigate pathogen transmission risks.

### 2.2 Species classification

Species identification was performed in the field based on external morphological characteristics, including body length, tail length, ear length and pelage colouration, following the taxonomic keys of Kingdon and Happold (2013) and Monadjem et al. (2015) (Supporting Information S2). Field identification was supplemented by molecular methods to confirm species identity for individuals identified as *Mastomys* sp., *Mus* sp., *Rattus* sp. and *Crocidura* sp. alongside a random subset of remaining individuals (50% of remaining samples).

Samples were stored at −20°C until processing. Genomic DNA was then extracted using QIAGEN DNeasy kits as per the manufacturer's instructions (QIAGEN, 2023) (Supporting Information S1). DNA extracts were amplified via polymerase chain reaction (PCR) using *cytochrome B* primers, and amplification success was confirmed via gel electrophoresis (Bangura et al., 2021). Full details of the PCR and sequencing protocols are provided in Supporting Information S1. Successful PCR products were purified and submitted for Sanger sequencing (performed by Eurofins Genomics). Attribution of sequences to rodent species was performed using the BLAST programme, comparing obtained sequences to *cytochrome B* records in the NCBI database (accessed 2023‐06‐30) (Altschul et al., 1990).

### 2.3 Description of small‐mammal detection and species community structure

Adequacy of sampling effort was assessed using species accumulation curves produced for each village study site and each land use type within a village study site, generated with the specaccum function from the vegan package (Oksanen et al., 2022). The rarefaction method was applied to evaluate whether sampling was sufficient to detect the expected rodent species within these categories. Curves were visually inspected, and most demonstrated a plateau, suggesting adequate sampling effort (Figure S3).

Detection/non‐detection histories for each grid cell and small‐mammal species were constructed, assigning ‘1’ when a species was detected and ‘0’ otherwise. Species communities were described at multiple spatial scales: (i) all species identified across all village sites and land use types, (ii) all species identified within a village study site and (iii) all species identified within a single land use type within a single village study site. Species richness and Shannon diversity were calculated at each of these spatial scales (Shannon, 1948).

### 2.4 Estimating the effect of land use on species occurrence and richness

To adjust for differential probabilities of detection, we used a Bayesian spatial latent factor multi‐species occupancy model that incorporates residual species correlations, imperfect detection, and spatial autocorrelation. The model assumes that the true occupancy state of a species at a site ($z_{i,j}$) is constant throughout the study period. Variable selection was informed by a pre‐specified conceptual model (Figure S4). Models were defined using the sfMsPGOcc function in the spOccupancy package in the R statistical computing language (Doser et al., 2022). This approach defines the true presence or absence (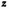) of a species (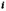), at grid cell (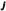) as arising from a Bernoulli process (Equation 1). Where $\psi_{i,j}$ is the probability of occurrence of a species at a grid cell. This is modelled using a logit link where $\beta_{i}$ are the species‐specific regression coefficients of the site‐specific covariates ($x_{j}^{⊤}$) and a latent process $w_{i,j}^{*}$ that incorporates residual species correlations and spatial autocorrelation (Equation 2).

(1) $$z_{i,j}∼\text{Bernoulli}\left(\psi_{i,j}\right)$$

(2) $$\text{logit}\left(\psi_{i,j}\right)=x_{j}^{⊤}\beta_{i}+w_{i,j}^{*}$$

The species‐specific regression coefficients ($\beta_{i}$) are specified as random effects arising from a common community‐level distribution (Equation 3), where $\mu_{\beta }$ is the community‐level mean effect and $T_{\beta }$ is a diagonal matrix representing the inter‐species variability.

(3) $$\beta_{i}∼\text{Normal}\left(\mu_{\beta },T_{\beta }\right)$$

The detection component estimates the unobserved $z_{i,j}$. Here, $y_{i,j,k}$ is the observed detection or non‐detection of a species 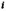, at site 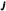, during replicate 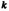 (Equation 4). This is approached as a Bernoulli process conditional on the true latent occurrence state $z_{i,j}$ and the detection probability $p_{i,j,k}$. The probability of a species being detected at a grid cell during a replicate (given it is present), is a function of grid cell and replicate specific covariates v and a set of species‐specific regression coefficients $\alpha_{i}$ (Equation 5).

(4) $$y_{i,j,k}∼\text{Bernoulli}\left(p_{i,j,k}z_{i,j}\right)$$

(5) $$\text{logit}\left(p_{i,j,k}\right)=v_{i,j,k}^{⊤}\alpha_{i}$$

Similarly to the occurrence component, these detection coefficients are specified as random effects arising from a common community‐level distribution (Equation 6).

(6) $$\alpha_{i}∼\text{Normal}\left(\mu_{\alpha },T_{\alpha }\right)$$

Minimally informative priors were specified for community‐level mean parameters ($\mu_{\alpha }$ and $\mu_{\beta }$, normal(0, 2.72)) and variance parameters ($T_{\alpha }$ and $T_{\beta }$, inverse‐gamma(0.1,0.1)).

We developed a set of candidate models based on our conceptual model (Figure S4) after assessing covariates for collinearity (correlation >0.8). Continuous variables were standardized. The full candidate model for occurrence included land use type, village, distance to permanent structure and elevation (Equation 7), while the full detection model included monthly precipitation, moon fraction, and trapping effort (Equation 8). The full detection model (Equation 8) was selected a priori based on theoretical support for these covariates and was used for all candidate occupancy models; therefore, model selection was performed on the occupancy component only.

(7) $$\begin{array}{c} \text{Probability of occurrence}∼\text{Land}\;\mathrm{use}\;\text{type}+\text{Village}\\ \;+\text{scale}\left(\text{Distance to permanent structure}\right)\\ \;+\text{scale}\left(\text{Elevation}\right) \end{array}$$

(8) $$\begin{array}{c} \text{Probability of detection}∼\text{scale}\left(\text{Monthly precipitation}\right)\\ \;+\text{Moon fraction}+\text{scale}\left(\text{Number of trap nights}\right) \end{array}$$

We first compared the candidate occupancy models within a spatial framework using the widely applicable information criterion (WAIC) (Table S1) (Watanabe, 2010). This initial comparison indicated that a model including landuse and village as main effects was the most parsimonious and best‐supported structure. However, subsequent diagnostic checks of all fitted spatial models revealed that the spatial random effect parameter (*ϕ*) had failed to converge (Rhat ≫ 1.0), indicating that there was insufficient residual spatial autocorrelation in the data to reliably estimate this parameter after accounting for the strong fixed effects of land use and village.

Consequently, to ensure the reliability of our inferences, we fitted a final, more parsimonious non‐spatial multi‐species occupancy model (MsPGOcc) using the best‐supported covariate structure (~landuse + village). This final non‐spatial model demonstrated excellent convergence for all key parameters (all Rhat < 1.15; WAIC = 4692.23) and was used for all subsequent analyses and interpretation.

Several candidate models demonstrated similarly strong performance (ΔWAIC < 4), indicating a degree of model selection uncertainty. Therefore, based on the principle of parsimony, we selected the simplest model from this top‐performing set for all subsequent inference. This final model included land use type and study village as main effects in the occurrence component (Equation 9). A model with an interaction between land use and the rural/peri‐urban classification (the group_landuse variable in Table S1) was also considered, but the main effects model provided a better, more parsimonious fit. For visualisation (Figure 3), we present the predictions from this final model stratified by the rural/peri‐urban classification to explore how these main effects manifest across this demographic gradient. Posterior predictive checks were used to assess the goodness‐of‐fit for the final model.

(9) Probability of occurrence∼Landusetype+Village

### 2.5 Co‐occurrence of *Mastomys natalensis* with sympatric species

To investigate the potential for biotic interactions to shape species distributions, we adopted a fully Bayesian approach to propagate the uncertainty from our final non‐spatial occupancy model into the co‐occurrence analysis. This method analyses the residual associations after accounting for environmental drivers and is designed to distinguish potential biotic interactions from patterns caused by shared habitat filtering (D'Amen et al., 2018).

The analysis was stratified by land use type. For each of the 2100 posterior samples from the model, we calculated the Spearman's rank correlation coefficient (*ρ*) between the vectors of occupancy probabilities (*ψ*) for each species pair across the relevant village strata (*N* = 3 for forest, *N* = 4 for agriculture and village). This process resulted in a full posterior distribution for *ρ* for each species pair within each land use type.

From this distribution, we calculated the median *ρ* and its 95% credible interval (CrI). An association was considered statistically robust if its 95% CrI did not overlap zero. In our interpretation, we focused on associations that were both statistically robust and demonstrated at least a moderate effect size (|median *ρ*| > 0.4). This analysis was restricted to species pairs that were empirically co‐detected within a given land use type. We interpret these robust correlations as strong evidence of spatial association. However, we acknowledge that this analysis does not allow for inferences regarding the causal mechanism or directionality of any observed relationships (e.g. whether one species excludes or is excluded by another) (Blanchet et al., 2020).

## 3 RESULTS

### 3.1 Small‐mammal detection and species community structure

Over the study period, 684 individual small mammals were detected from 43,266 trap nights across the four village study sites, yielding an overall trap success (TS) of 1.6%. Species accumulation curves suggest that sampling effort was generally adequate, although some curves, particularly for land use types within a single village, did not reach a clear plateau, indicating that additional rare species might be detected with further sampling (Figure S3).

The agricultural areas had the highest species richness and Shannon diversity values, while trap success was greatest in village settings (Table 1). Among the study sites, Seilama had the highest overall trap success and species richness. Notably, Seilama also exhibited the greatest trap success within agricultural areas, unlike the other three sites. Species richness in Seilama was twice that of Lambayama, the peri‐urban village study site, which had the lowest species richness and Shannon diversity. In Lambayama, most rodents were detected within built‐up village areas, reflecting its proximity to Kenema city.

**TABLE 1:** The number of trapped individuals (*N*), the number of trap nights (TN), trap success (TS %), species richness and Shannon diversity by village and land use type.

Village	Land use	*N*	TN (TS %)	Species richness	Shannon diversity
Baiama	Village	73	2716 (2.7%)	8	1.11
	Agriculture	45	4696 (1%)	9	1.96
	Forest	3	1568 (0.2%)	2	0.64
	Total	121	8980 (1.3%)	12	2.18
Lalehun	Village	54	2824 (1.9%)	9	1.65
	Agriculture	98	7608 (1.3%)	13	2.18
	Forest	5	1862 (0.3%)	3	1.05
	Total	157	12,294 (1.3%)	13	2.73
Lambayama	Village	93	2736 (3.4%)	4	0.42
	Agriculture	50	6260 (0.8%)	6	1.19
	Total	143	8996 (1.6%)	6	1.34
Seilama	Village	41	3240 (1.3%)	8	1.54
	Agriculture	186	7836 (2.4%)	13	1.97
	Forest	36	1920 (1.9%)	8	1.51
	Total	263	12,996 (2%)	14	2.65
All villages	Village	261	11,516 (2.3%)	12	2.38
	Agriculture	379	26,400 (1.4%)	17	3.14
	Forest	44	5350 (0.8%)	10	1.99
	Total	684	43,266 (1.6%)	17	3.65

**FIGURE 2:**
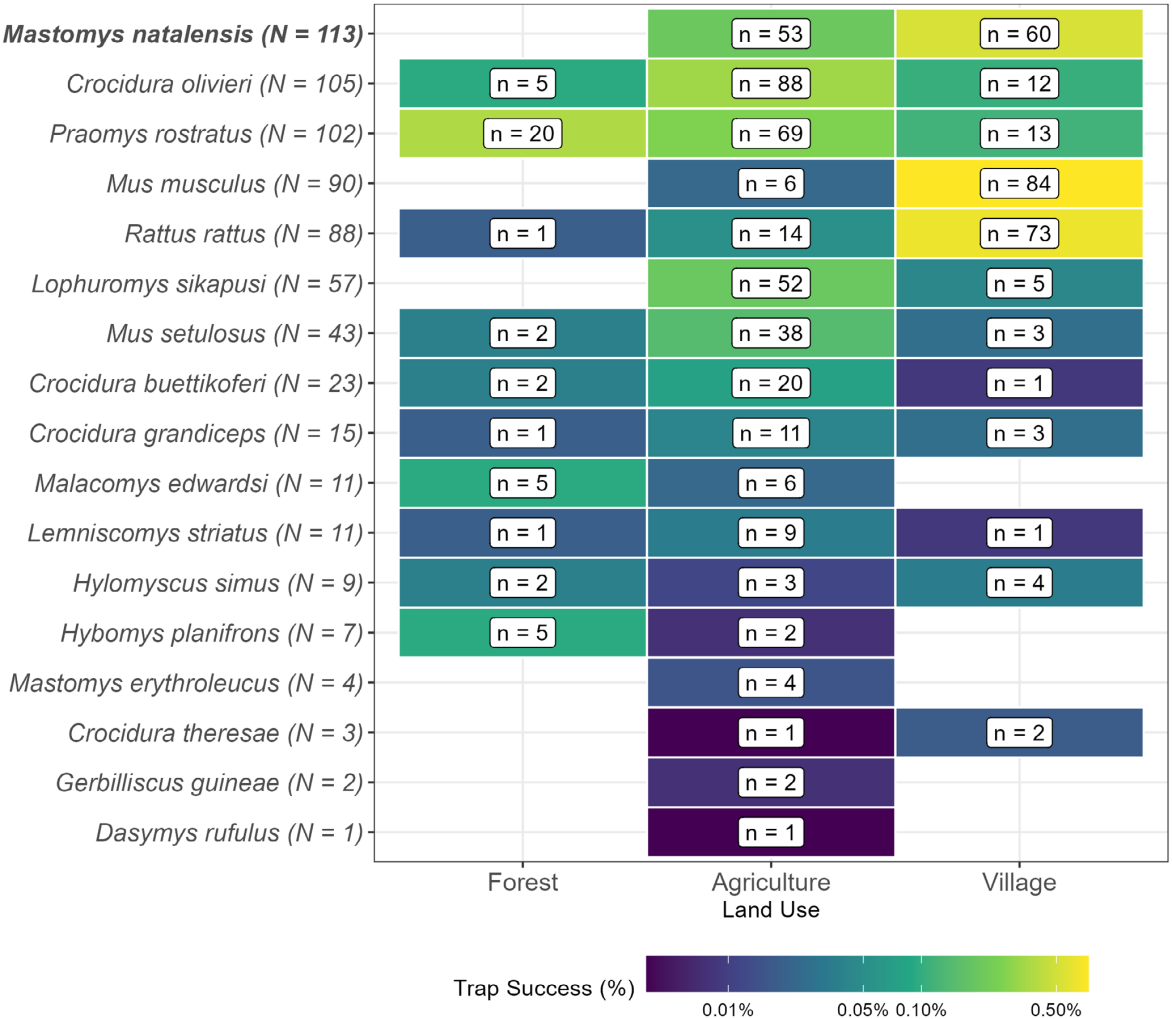
Trap success of small mammal species across different land use types (grouped across village study sites). The total number of detections in this study is shown next to the species binomial name (*N*). The number of detections of each species in each land use is shown in the label (*n*). The colour of the tile corresponds to the trap success. Mastomys natalensis is bolded to highlight its status as the primary reservoir host of LASV.

The most commonly detected rodent species across all land use types was *M. natalensis* (*N* = 113, 16.5%), followed by *Praomys rostratus* (*N* = 102, 14.9%), *M. musculus* (*N* = 90, 13.2%), *R. rattus* (*N* = 88, 12.9%), *Lophuromys sikapusi* (*N* = 57, 8.3%) and *Mus setulosus* (*N* = 43, 6.3%). Among non‐rodent species, the insectivorous shrew *Crocidura olivieri* was the most frequently detected (*N* = 105, 15.4%). *Mastomys natalensis* and *R. rattus* were found in all village study sites, although *M. natalensis* was absent in forest settings (Figure 2). The invasive rodent *M. musculus* was detected only in Lambayama, the peri‐urban site.

Trap success varied significantly by species, land use type, and village study site. The highest TS was observed for *M. musculus* in the Lambayama village site, while *M. natalensis* and *R. rattus* showed high TS across multiple sites in village land use types. In contrast, *P. rostratus* had the highest TS in forest and agricultural areas.

Seasonal variation in trap success was not consistent across all species. For instance, *M. musculus* and *L. sikapusi* were detected more frequently in the dry season, while for most other species, trap success was similar between seasons (Figure S5A). When stratified by land use type, more distinct variations were evident. For instance, *M. natalensis* had higher TS in village settings than in agricultural areas during the rainy season, while TS in these two habitats were more similar during the dry season. *P. rostratus* showed substantially higher TS in forests during the dry season compared to the rainy season. Other species showed notable seasonal patterns: TS for *R. rattus* were higher in villages than in agriculture in both seasons, with this difference being more pronounced during the rainy season. Finally, *L. sikapusi* TS was highest in agricultural areas during the dry season but shifted to being higher in villages during the rainy season (Figure S5B).

### 3.2 Estimating the effect of land use on species occurrence and richness

**FIGURE 3:**
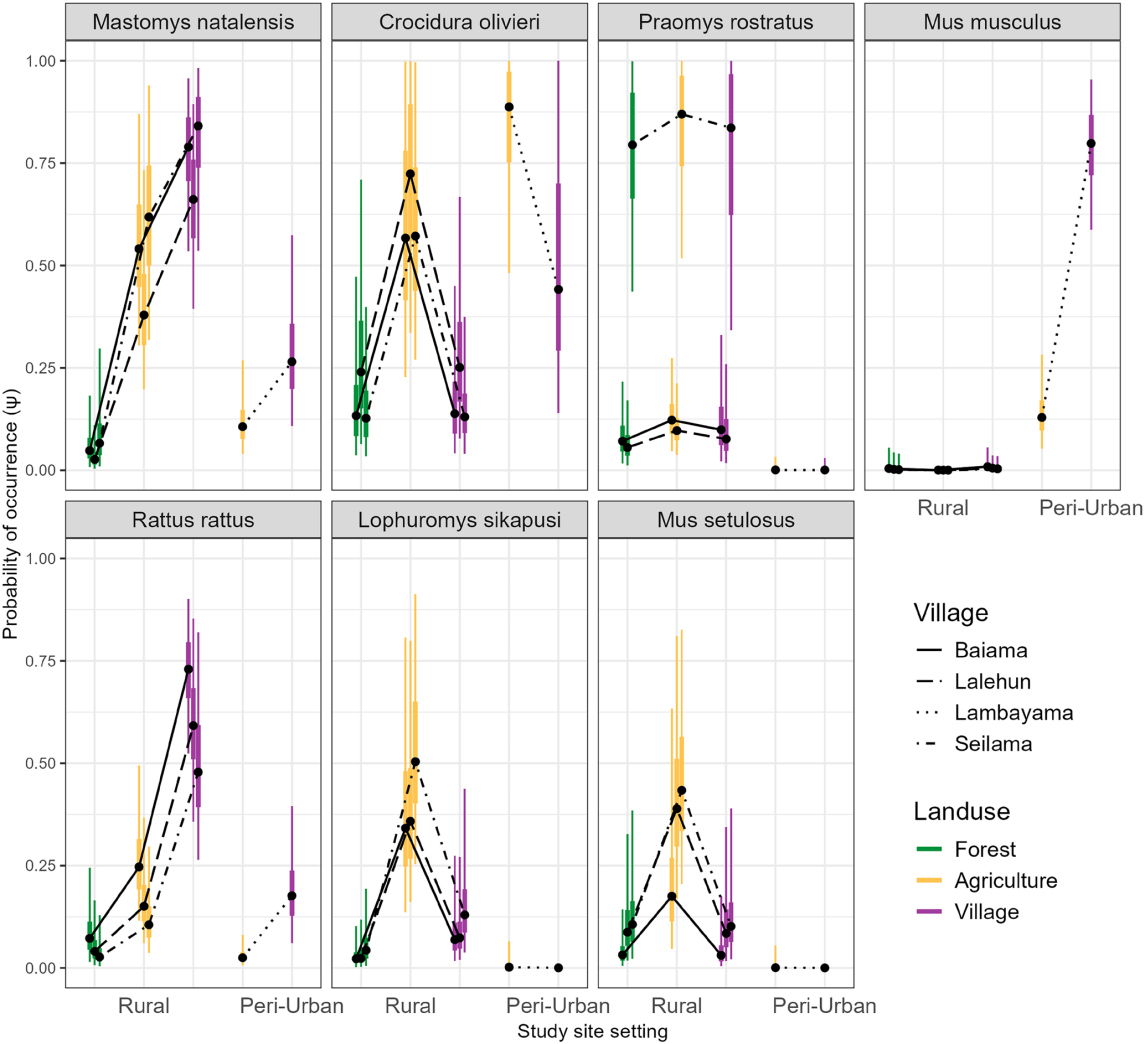
Probability of species occurrence (ψ) across a land use gradient, stratified by rural and peri‐urban village study sites. The visualization shows the posterior distributions from the final non‐spatial occupancy model for each unique combination of covariates. Thick error bars represent the 50% credible interval (the interquartile range of the posterior), and thin lines represent the 95% credible interval. Black points indicate the posterior median. Lines connect the medians for each village to illustrate site‐specific patterns.

We found several distinct patterns in the probability of occurrence (ψ) within a trapping grid cell for the seven included species (Figure 3 and, marginal effects of the detection parameters are shown in Figures S6–S8). First, the synanthropic species, *M. natalensis*, *R. rattus* and *M. musculus* had their greatest probabilities of occurrence in villages. However, *M. natalensis* differed from the two invasive species (*R. rattus* and *M. musculus*) as its probability of occurrence in agricultural settings, remained generally high. Second, the distribution of *P. rostratus* was strongly site‐specific, it had very high occupancy probabilities across all lad use types in one rural village (Seilama) but much lower probabilities in others. Finally, *C. olivieri*, *L. sikapusi* and *M. setulosus* generally had their highest probabilities of occurrence in agricultural areas with lower and more similar probabilities in forests and villages. No species showed high probability of occurrence across all land use types, consistent with species being adapted to distinct ecological niches. The strong influence of the village covariate in our final model indicates that unmeasured village‐level factors likely contribute significantly to this observed variation.

To further explore this, we stratified the results by the rural and peri‐urban classification (Figure 3). The probability of occurrence of *M. natalensis* was substantially lower in the peri‐urban study site compared to the rural sites. The same pattern was observed for *R. rattus*. For native rodent species such as *P. rostratus* and *L. sikapusi*, occupancy probabilities were consistently higher across all land uses in rural sites compared to the peri‐urban site, where they were nearly absent. The shrew *C. olivieri*, however, showed a different pattern, with a higher probability of occurrence in the peri‐urban site, particularly in agriculture, compared to the rural sites.

In contrast to species found throughout our study area, *M. musculus* was predicted to have a low probability of occurrence in all land use types in rural areas, with high probabilities of occurrence only for village settings in the single peri‐urban site. The occurrence probabilities for the three commensal species (*M. natalensis*, *R. rattus* and *M. musculus*) suggest that competition may be a factor in reducing the occurrence probabilities of *M. natalensis* and *R. rattus* in the presence of *M. musculus*, as in its absence these two species have high occurrence probabilities in villages.

### 3.3 Co‐occurrence of species within land use types

**FIGURE 4:**
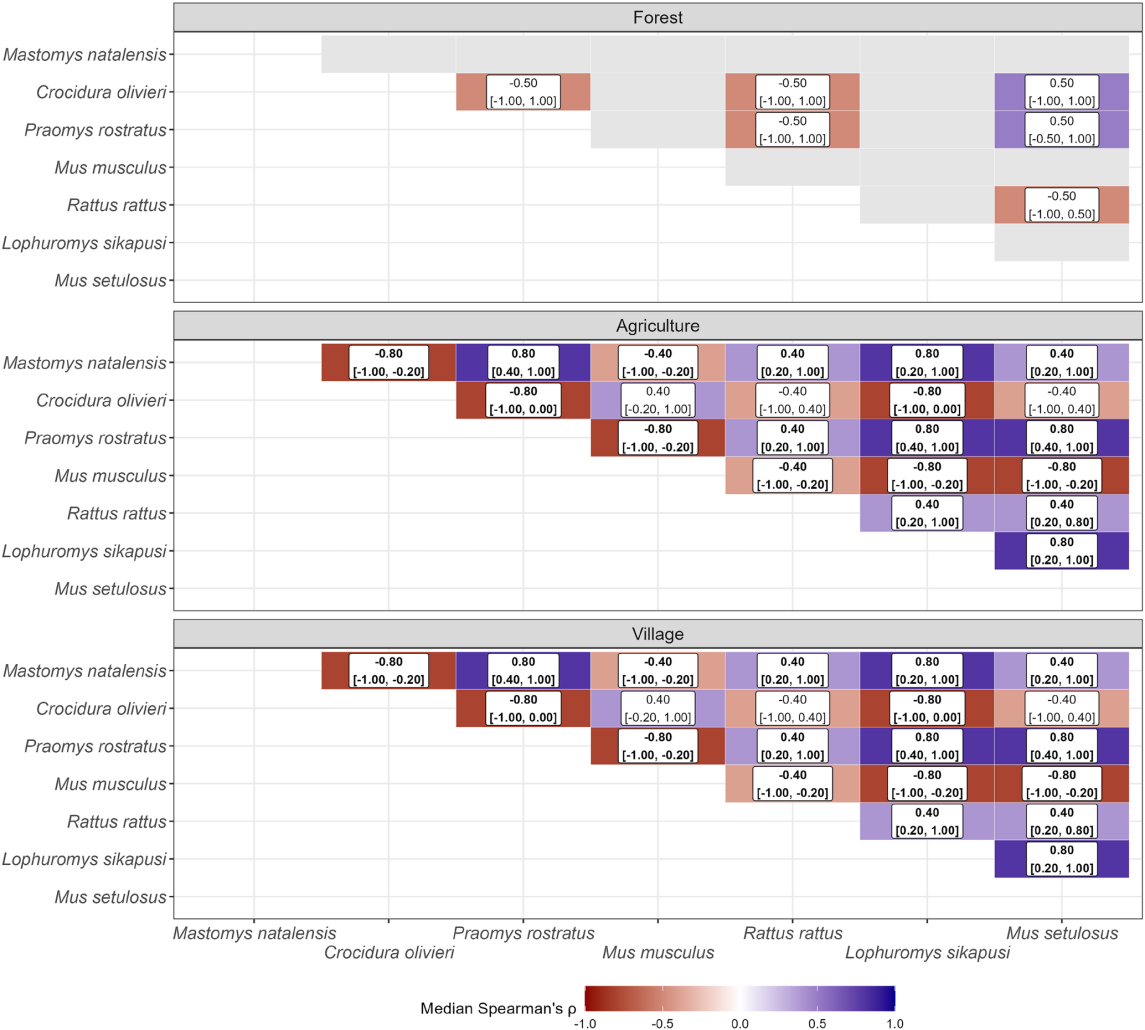
Posterior summary of Spearman's rank correlations (*ρ*) for species' occupancy probabilities, calculated across village strata within each land use type. The colour of each tile represents the median of the posterior distribution for the correlation coefficient. The text in each cell displays the median *ρ* followed by the 95% Credible Interval [lower, upper]. Labels in bold indicate associations that are both statistically robust (95% CrI does not overlap zero) and ecologically meaningful (|median *ρ*| > 0.4). Grey tiles indicate species pairs that were never empirically co‐detected in a given land use and were therefore excluded from the analysis.

The analysis of the posterior distributions for Spearman's rank correlation coefficients reveals patterns consistent with our original hypothesis that the local spatial distribution of *M. natalensis* is regulated by biotic interactions (Figure 4). In human‐dominated landscapes, we found strong evidence for several associations. For *M. natalensis* and the invasive *M. musculus*, there was a robust negative correlation in both agricultural (median *ρ* = −0.4, 95% CrI [−1.00, −0.20]) and village (median *ρ* = −0.4, 95% CrI [−1.00, −0.20]) habitats. In contrast, a robust positive correlation was found between *M. natalensis* and the other invasive rodent, *R. rattus*, in both agricultural (median *ρ* = 0.4, 95% CrI [0.20, 1.00]) and village (median *ρ* = 0.4, 95% CrI [0.20, 1.00]) settings.

Beyond these focal species, other strong patterns of association were evident. The presence of *M. musculus* was strongly and negatively correlated with the entire native rodent guild (*P. rostratus*, *L. sikapusi* and *M. setulosus*) in both agricultural and village habitats. Conversely, these native rodent species were all strongly and positively correlated with one another in the same habitats, suggesting a cohesive community structure. A different pattern was observed for the insectivorous shrew, *C. olivieri*, which was robustly and negatively correlated with most rodent species in agricultural and village settings. In the forest habitat, however, while some median correlations appeared strong, the posterior uncertainty was very high; the 95% credible intervals for all species pairs overlapped zero, indicating a lack of statistically robust evidence for any associations in that land use type.

## 4 DISCUSSION

This study provides valuable insights into the dynamics of small‐mammal communities in a Lassa fever endemic region of Eastern Sierra Leone, focussing on how land use types influence species richness, diversity and the risk of viral spillover. Our results indicate that species richness and diversity were highest in agricultural settings. These habitats support both synanthropic and non‐synanthropic species, increasing the potential for viral sharing. The highest probability of occupancy for the main LASV reservoir host, *M. natalensis*, was observed in villages and agricultural settings, while it was likely absent from forests. This pattern mirrors that of the invasive commensal species *M. musculus* and *R. rattus*, suggesting that human‐modified landscapes are key drivers of rodent distribution. Stratification by human population density revealed that *M. natalensis* occupancy was lower in the peri‐urban site, where *M. musculus* dominates. Importantly, we identified a negative correlation in the probability of co‐occurrence between *M. natalensis* and *M. musculus*, which could have important implications for understanding observed Lassa fever outbreaks.

### 4.1 Small‐mammal communities are associated with land use type

Small‐mammal species richness was greatest in agricultural settings where both synanthropic and non‐synanthropic species were found. The higher species richness in agricultural environments provides more opportunities for cross‐species transmission of LASV within diverse small‐mammal communities.

Evidence suggests that the wider species community may play a role in LASV transmission. While *M. natalensis* is the primary reservoir, acute LASV infection (confirmed by viral RNA or culture) has been documented in five other rodent species: *Hylomyscus pamfi*, *Mastomys erythroleucus*, *Mus baoulei*, *Mus minutoides* and *Rattus rattus* (Table S2A) (Demby et al., 2001; Fichet‐Calvet et al., 2014; Monath et al., 1974; Olayemi, Cadar, et al., 2016; Yadouleton et al., 2019). However, the evidence for *M. minutoides* and *R. rattus* comes from a single 1975 study where species were identified by morphology alone, and these findings should be interpreted with the understanding that they lack modern genetic confirmation (Wulff et al., 1975). Broader exposure within small mammal communities is suggested by serological surveys, which have detected LASV‐reactive antibodies in 10 further rodent species (*Arvicanthis nilotics*, *Gerbilliscus kempii*, *Lemniscomys striatus*, *Lophuromys sikapusi*, *Malacomys edwardsi*, *Mus musculus*, *Mus setulosus*, *Praomys daltoni*, *Praomys misonnei* and *Praomys rostratus*) and one shrew genus (*Crocidura*) (Table S2B) (Simons et al., 2025). Notably, this includes serological evidence for both *M. minutoides* and *R. rattus*, which may lend some support to the earlier acute infection findings. However, these serological results must also be interpreted with caution. The detection of antibodies confirms exposure to an arenavirus but depending on assay sensitivity and specifity they may not be solely antibodies to LASV; given the increasing discovery of novel arenaviruses in West African small mammals, some seropositivity, particularly in genera like *Arvicanthis* and *Gerbilliscus*, could reflect exposure to other, related viruses (e.g. Ippy virus) rather than LASV itself (Saluzzo et al., 1988).

Whether these infections are incidental or represent competent chains of viral transmission remains unclear. It is possible that viral sharing is greatest in the more species‐rich agricultural settings, allowing introduction or re‐introduction of LASV into isolated commensal species populations following local viral extinction (Bordes et al., 2015). This may be particularly important for maintaining viral persistence through time in spatially isolated *M. natalensis* populations, where rapid depletion of susceptible individuals might be expected (Goyens et al., 2013). Our findings that *M. natalensis* is absent from forested regions may indicate that its populations are spatially isolated, particularly in the mosaic landscapes of remnant forest and agriculture that characterize much of West Africa (Denys et al., 2005; Leirs, Verheyen, & Verhagen, 1996; Mariën et al., 2018). The role of the wider rodent community in facilitating LASV transmission between these isolated populations warrants further investigation.

Although previous studies from Guinea and Sierra Leone have reported seasonal fluctuations in *M. natalensis* abundance, our findings of similar or increased abundance during the dry season suggest that localized factors, such as agricultural practices or food storage, may influence rodent behaviour in this region differently than previously documented (Bangura et al., 2021; Fichet‐Calvet et al., 2007). Alternatively, increased trap‐shyness during periods of increased abundance may mask replication of previous findings. Further studies on small‐mammal communities, incorporating local human community behaviour and practices, conducted over longer time periods and across different geographic regions, would be valuable in identifying how seasonal habitat preferences of these rodents vary (Kelly et al., 2013; Leach et al., 2017). These findings underscore the importance of considering local human activities, such as food storage and agricultural practices, when modelling seasonal patterns in rodent behaviour. Such practices may alter rodent abundance and movement in ways that differ from areas where *M. natalensis* displays more predictable seasonal patterns due to other environmental or ecological factors.

The abundance of *M. natalensis* within households has been suggested as a key driver of Lassa fever outbreaks, likely due to increased human–rodent contact (Bonwitt et al., 2017; Mariën et al., 2020). Therefore, the movement of *M. natalensis* from species‐rich agricultural areas to households may play a critical role in the transmission dynamics of LASV.

### 4.2 Evidence for biotic interactions shaping patterns of small‐mammal species diversity

The segregation of small‐mammal species into distinct ecological niches, categorized by human‐dominated or non‐human‐dominated land use types, underscores the significant role of abiotic factors in shaping species distributions. However, within these habitats, the residual spatial associations suggest that biotic interactions also play a key role. While recognising the inherent challenges of inferring ecological processes from spatial patterns, our analysis of residual correlations after accounting for habitat effects reveals several important associations (Blanchet et al., 2020).

Our findings show a high predicted occupancy of both *M. natalensis* and *R. rattus* in human‐dominated landscapes, with a positive correlation in their co‐occurrence, aligning with a similar study in Sierra Leone (Bangura et al., 2021). In contrast, the negative correlation between *M. musculus* and the larger *M. natalensis* and *R. rattus* in the peri‐urban site presents an apparent competitive paradox. Direct physical competition seems unlikely given the significant size difference. However, evidence from Senegal suggests that *M. musculus* can indeed displace both native rodents and *R. rattus* during its expansion (Dalecky et al., 2015). The mechanism is likely not direct aggression but rather a combination of competition, niche specialisation and effects of predation (Polis & Holt, 1992). *Mus musculus* is exceptionally adapted to the indoor environment and may be a more efficient forager on the scattered, high‐quality resources found within human dwellings in West Africa (Vrbanec et al., 2021). Coupled with a high reproductive rate, it may be able to numerically saturate this specific niche, effectively excluding more generalist species like *M. natalensis* that utilise both indoor and surrounding outdoor habitats. Furthermore, the invasion dynamics themselves may play a role. Dalecky et al. (2015) describe separate invasion fronts for *M. musculus* and *R. rattus*, suggesting that once one species establishes dominance in a locality via a particular human transport route, it may create a biotic barrier that resists invasion by the other. This would explain the mutually exclusive patterns observed in our study and others (Eskew et al., 2024).

Beyond the focal interactions involving invasive species, the analysis revealed broader community‐level structures that were highly context‐dependent. The strongest evidence for biotic associations was found in human‐dominated landscapes. In both agricultural and village habitats, the native rodent guild, including *P. rostratus*, *L. sikapusi* and *M. setulosus*, showed robust positive correlations with one another, suggesting a cohesive community. In stark contrast, the presence of *M. musculus* was strongly and negatively correlated with this entire native guild in these same habitats. This pattern indicates that the establishment of the house mouse may lead not just to the displacement of a single competitor, but to a wholesale perturbation of the native rodent community. In the forest, however, while the median correlations suggested some associations, the posterior uncertainty was very high for all species pairs, and we found no statistically robust evidence for co‐occurrence patterns in this land use type. A different pattern of segregation was observed for the insectivorous shrew, *C. olivieri*, which was generally negatively correlated with most rodent species, likely reflecting niche partitioning between trophic guilds. We interpret this finding with caution, as our grain‐based bait may have been less directly attractive to shrews; however, insects drawn to the bait could have indirectly attracted these insectivores, creating a complex detection scenario.

### 4.3 Benefits and challenges of systematic small‐mammal community sampling

Systematic investigation of small‐mammal communities entails a greater sampling effort than targeted sampling of specific species in selected habitats. In this study, the overall TS was relatively low compared to other studies focusing on synanthropic rodent species (Bangura et al., 2021; Happi et al., 2022; Olayemi et al., 2018). Our TS of 2.3% within villages aligns with the 3% reported in Bo, Sierra Leone, but is notably lower than the 17% and 14% reported in Nigeria and Guinea, respectively (Fichet‐Calvet et al., 2007; Happi et al., 2022). This discrepancy may arise from differences in trapping methodologies, including the size of traps, trap locations, or the behaviour of target species. For example, *M. natalensis* captures in its Western range were less frequent than those observed in Tanzania, where TS of around 24% were reported in agricultural settings (Mulungu et al., 2013). One potential explanation is that food availability in each setting might affect the species' behaviour, with environmental food sources influencing trap shyness (Stryjek et al., 2019; Taylor et al., 1974).

Despite the higher sampling effort required, systematic small‐mammal community sampling offers distinct advantages over targeted species sampling. By adopting a broader, more inclusive approach, this method reduces the risk of overlooking less common species that may act as pathogen hosts and provides greater insight into biotic interactions between species. For instance, our sampling design allowed us to observe associations between synanthropic species like *M. musculus* and native species, offering a more holistic view of the ecological factors contributing to pathogen spread.

Comparison between studies using different sampling techniques presents several challenges. Previous studies on rodent communities in the Lassa fever endemic region have used TS as an indirect measure of rodent abundance (Bangura et al., 2021; Fichet‐Calvet et al., 2009; Olayemi et al., 2018). While we also present TS as a descriptive metric, we acknowledge that it can be a biased proxy for abundance. Our analysis, incorporating a model of imperfect detection, suggests estimating abundance from TS may not be applicable across different land use types and species (Figure S8). For example, we observed that the probability of detecting *M. musculus* and *R. rattus* was higher than for native species when a consistent amount of trapping effort was applied. This finding aligns with previous research showing that trap success may be an unreliable proxy for relative abundance (Parsons et al., 2022). Therefore, improving harmonisation of rodent sampling designs could enhance direct comparisons of species communities and pathogen prevalence across the Lassa fever endemic region (Simons et al., 2023).

There are several limitations to the current study. One limitation is the relatively short duration of our animal sampling (less than 3 years). Given that rodent populations can fluctuate significantly over longer time periods, it is possible that important variations in abundance were missed. As a result, the probability of occurrence for certain species may have been underestimated, particularly for species that were at low abundance during our survey period. Extending the sampling period would help to better capture temporal changes in species occurrence and abundance. Furthermore, our trapping protocol, which involved closing traps during the day, likely under‐represents diurnal or crepuscular species such as *L. striatus*, which could affect our estimates of community composition and diversity. Additionally, land use in Sierra Leone, particularly in agricultural areas, follows multi‐year cycles of cultivation, fallow and reconversion to cultivated land. To more comprehensively understand the effects of land use change on rodent communities, it would be useful to focus on a single location that spans the transition from forested land to agricultural, fallow and degraded forest land use. Such a study would provide insights into how these different land management practices influence rodent community dynamics and pathogen risk. Finally, unobserved characteristics of the villages in our study may have contributed to variations in the composition of rodent communities, as suggested by the wide posterior distributions for some species. Expanding our study to include a greater number of villages would help increase the generalisability of our findings.

### 4.4 Implications for understanding the risk of LASV spillover

The lower occurrence of *M. natalensis* in certain land uses is consistent with growing evidence of LASV prevalence heterogeneity across the endemic region (Mariën et al., 2020). In some village communities, no current evidence of LASV transmission has been detected within rodent populations, despite prior human cases or serological evidence of outbreaks. This suggests that pathogen prevalence in rodents may vary significantly over time and space (Bangura et al., 2021; Leski et al., 2015; McCormick et al., 1987). As discussed earlier, LASV transmission within a host community may be short‐lived, with rapid local extinction of the virus (Goyens et al., 2013). These dynamics raise the possibility that non‐*M. natalensis* species may play a crucial role in transferring the virus between communities of *M. natalensis* that are separated by unsuitable habitat, leading to pathogen re‐introduction.

Current disease models of LASV risk largely fail to incorporate the role of multiple rodent species and the potential biotic interactions between them (Basinski et al., 2021; Fichet‐Calvet & Rogers, 2009; Klitting et al., 2022; Mylne et al., 2015; Olugasa et al., 2014; Redding et al., 2016). Our findings, which highlight interactions between *M. natalensis* and primarily *M. musculus*, suggest that Lassa fever risk could potentially be reduced in areas where *M. musculus* is present. This raises the need for further research into the competence of *M. musculus* as a host for LASV, as previous serological evidence has shown exposure to the virus in this species (Demby et al., 2001). If *M. musculus* is not a competent host for LASV, this could help explain why Lassa fever is more commonly reported in rural areas of the endemic region, rather than urban centres, where *M. musculus* may have displaced more competent viral hosts. To test this hypothesis, further research systematically sampling along the urban–rural gradient is essential. Such work could significantly impact future projections of Lassa fever risk, especially given the rapid population growth and urbanisation occurring across West Africa.

## AUTHOR CONTRIBUTIONS

DS, RG, EF‐C, DW‐J, RK and KEJ conceived the ideas and designed the methodology. DS, UB, DiS, JL, JK, MJ, MD, JoL and RA collected the data. DS and RG analysed the data. DS, RG and KEJ interpreted the analysis. DS, UB, EF‐C, DW‐J, RK and KEJ provided supervision and resources to conduct the study. DS led the writing of the manuscript. RG, DW‐J, RK and KEJ contributed critically to drafts. All authors gave final approval for publication.

## CONFLICT OF INTEREST STATEMENT

All authors report no financial conflicts of interest.

## STATEMENT ON INCLUSION

This study brings together authors from several countries, including scientists based in the country where the study was carried out. All authors were engaged early on with the research and study design to ensure that the diverse sets of perspectives they represent were considered. Literature published by scientists from the study country and wider region was cited. Study protocols were discussed with local scientists for appropriateness of design. Consultations were held with community leaders of the study villages and the wider region before finalising the study design and enrolment of sites into the study.

## Supporting information


**Table S1:** Model comparison for the Bayesian spatial latent factor multi‐species occupancy model. Models are ordered by WAIC. ELPD, expected log pointwise predictive density, pD, the effective number of parameters, WAIC, widely applicable information criterion.
**Table S2:** Sampling of rodents for LASV.
**Figure S1:** Trap locations and number of trap nights in (A) Baiama, (B) Lalehun, (C) Lambayama and (D) Seilama. Each cell represents a 49 m^2^ area. The colour of cells relates to the trapping effort within that area in number of trap nights. The facets relate to the land use type of the trapping site. Images obtained from Google maps, copyright 2023 CNES/Airbus, Maxar Technologies.
**Figure S2:** Schematic diagram of conversion from trap locations to grids. (A) Individual traps were placed in a grid structure in a pre‐specified location. While attempts were made to keep trap locations over repeated visits individual traps were often placed in slightly different locations. This is shown in the schematic using different colours to represent different visits. (B) To harmonise the locations of traps to coordinates that could be used in the spatial occupancy model we aligned a regular grid with grid cell sizes of 49 m^2^ over the trapping area and assigned individuals traps to these cells. (C) The number of traps and therefore the number of trapnights within each grid cell was aggregated for each visit. The number of trap nights informed the detection component of the species occupancy model. Detection histories were produced for each grid cell that was sampled for each species. Grid cells were assigned a 1 if any trap within the grid cell detected the species and 0 otherwise.
**Figure S3:** (A) Species accumulation curves by village site. (B) Species accumulation curves by village site stratified by land use type. The *x*‐axis represents the number of unique 49 m^2^ grid cells (sites) accumulated, not individual trap nights.
**Figure S4:** Conceptual model used to identify potential causal pathways for inclusion of variables for the occupancy and detection model specification.
**Figure S5:** (A) Trap success for all species by season (rainy = May–October, dry = November–April) of trapping activity. (B) Trap success for all species by season (rainy = May–October, dry = November–April) stratified by land use type.
**Figure S6:** The marginal effect of mean monthly rainfall on the probability of detection of a species in a grid cell. The black line shows the mean modelled probability of detection for the amount of monthly rainfall, the shaded grey region represents the 95% credible interval (CrI). Probability of detection varies by species with higher values for the invasive rodent species *M. musculus* and *R. rattus*, than the native rodent species. There is a general response of decreasing probability of detection with increasing rainfall.
**Figure S7:** The marginal effect of the fraction of the full moon on the probability of detection of a species in a grid cell. The black line shows the mean modelled probability of detection for the moon phase, the shaded grey region represents the 95% CrI. Probability of detection varies by species with higher values for the invasive rodent species *M. musculus* and *R. rattus*, than the native rodent species. There is no important response to moon phase for most species. The probability of detection appears to fall for *M. natalensis* with increasing moon phase but the credible intervals overlap for the entire range.
**Figure S8:** The marginal effect of trapping effort (TN) on the probability of detection of a species in a grid cell. The black line shows the mean modelled probability of detection trapping effort, the shaded grey region represents the 95% CrI. Probability of detection is low for all species at low levels of TN. The probability of detection with increasing TN varies by species. The invasive rodent species *M. musculus* and *R. rattus* show a sinusoidal response over the range of TNs that were observed in this study with the probability of detection being greater than 50% at relatively low numbers of TN (17 and 22, respectively). A much greater trapping effort were required to obtain the same probability of detection for native rodent species. Only *M. natalensis* reached 50% detection among the range of TN conducted within grid cells in this study, requiring 29 TN to reach a probability of 50% detection.

## Data Availability

Data are available on PHAROS (https://pharos.viralemergence.org/projects/?prj=prjyg91YQvrdk). All R scripts to reproduce the analysis are available on GitHub (https://github.com/DidDrog11/land‐use‐lassa‐hosts).

## References

[jane70187-bib-0001] Agbonlahor, D. E. , Akpede, G. O. , Happi, C. T. , & Tomori, O. (2021). 52 years of Lassa fever outbreaks in Nigeria, 1969–2020: An epidemiologic analysis of the temporal and spatial trends. The American Journal of Tropical Medicine and Hygiene, 105, 974–985. 10.4269/ajtmh.20-1160 34460421 PMC8592130

[jane70187-bib-0002] Altschul, S. F. , Gish, W. , Miller, W. , Myers, E. W. , & Lipman, D. J. (1990). Basic local alignment search tool. Journal of Molecular Biology, 215(3), 403–410. 10.1016/S0022-2836(05)80360-2 2231712

[jane70187-bib-0003] Arruda, L. B. , Haider, N. , Olayemi, A. , Simons, D. , Ehichioya, D. , Yinka‐Ogunleye, A. , Ansumana, R. , Thomason, M. J. , Asogun, D. , Ihekweazu, C. , Fichet‐Calvet, E. , & Kock, R. A. (2021). The niche of one health approaches in Lassa fever surveillance and control. Annals of Clinical Microbiology and Antimicrobials, 20(1), 29. 10.1186/s12941-021-00431-0 33894784 PMC8067790

[jane70187-bib-0004] Bangura, U. , Buanie, J. , Lamin, J. , Davis, C. , Bongo, G. N. , Dawson, M. , Ansumana, R. , Sondufu, D. , Thomson, E. C. , Sahr, F. , & Fichet‐Calvet, E. (2021). Lassa virus circulation in small mammal populations in Bo District, Sierra Leone. Biology, 10(1), 28. 10.3390/biology10010028 33466234 PMC7824740

[jane70187-bib-0005] Basinski, A. J. , Fichet‐Calvet, E. , Sjodin, A. R. , Varrelman, T. J. , Remien, C. H. , Layman, N. C. , Bird, B. H. , Wolking, D. J. , Monagin, C. , Ghersi, B. M. , Barry, P. A. , Jarvis, M. A. , Gessler, P. E. , & Nuismer, S. L. (2021). Bridging the gap: Using reservoir ecology and human Serosurveys to estimate Lassa virus spillover in West Africa. PLoS Computational Biology, 17(3), e1008811. 10.1371/journal.pcbi.1008811 33657095 PMC7959400

[jane70187-bib-0006] Blanchet, F. G. , Cazelles, K. , & Gravel, D. (2020). Co‐occurrence is not evidence of ecological interactions. Ecology Letters, 23(7), 1050–1063. 10.1111/ele.13525 32429003

[jane70187-bib-0007] Bonner, P. C. , Schmidt, W.‐P. , Belmain, S. R. , Oshin, B. , Baglole, D. , & Borchert, M. (2007). Poor housing quality increases risk of rodent infestation and Lassa fever in refugee camps of Sierra Leone. The American Journal of Tropical Medicine and Hygiene, 77(1), 169–175. 17620650

[jane70187-bib-0008] Bonwitt, J. , Sáez, A. M. , Lamin, J. , Ansumana, R. , Dawson, M. , Buanie, J. , Lamin, J. , Sondufu, D. , Borchert, M. , Sahr, F. , Fichet‐Calvet, E. , & Brown, H. (2017). At home with Mastomys and Rattus: Human‐rodent interactions and potential for primary transmission of Lassa virus in domestic spaces. The American Journal of Tropical Medicine and Hygiene, 96(4), 935–943. 10.4269/ajtmh.16-0675 28167603 PMC5392645

[jane70187-bib-0009] Bordes, F. , Blasdell, K. , & Morand, S. (2015). Transmission ecology of rodent‐borne diseases: New Frontiers. Integrative Zoology, 10(5), 424–435. 10.1111/1749-4877.12149 26176684

[jane70187-bib-0010] Carlson, C. J. , Brookson, C. B. , Becker, D. J. , Cummings, C. A. , Gibb, R. , Halliday, F. W. , Heckley, A. M. , Huang, Z. Y. X. , Lavelle, T. , Robertson, H. , Vicente‐Santos, A. , Weets, C. M. , & Poisot, T. (2025). Pathogens and planetary change. Nature Reviews Biodiversity, 1(1), 32–49. 10.1038/s44358-024-00005-w PMC7618822 41789152

[jane70187-bib-0011] Cuypers, L. N. , Cuypers, W. L. , Gildemyn‐Blomme, A. , Abraham, L. , Aertbeliën, S. , Massawe, A. W. , Borremans, B. , Gryseels, S. , & Leirs, H. (2017). No evidence for avoidance of black rat scent by the presumably less competitive natal multimammate mouse in a choice experiment. African Zoology, 52(2), 119–123. 10.1080/15627020.2017.1307139

[jane70187-bib-0012] Dalecky, A. , Ba, K. , Piry, S. , Lippens, C. , Diagne, C. A. , Kane, M. , Sow, A. , Diallo, M. , Niang, Y. , Konečný, A. , Sarr, N. , Artige, E. , Charbonnel, N. , Granjon, L. , Duplantier, J.‐M. , & Brouat, C. (2015). Range expansion of the invasive house mouse *Mus musculus domesticus* in Senegal, West Africa: A synthesis of trapping data over three decades, 1983–2014. Mammal Review, 45(3), 176–190. 10.1111/mam.12043

[jane70187-bib-0013] D'Amen, M. , Mod, H. K. , Gotelli, N. J. , & Guisan, A. (2018). Disentangling biotic interactions, environmental filters, and dispersal limitation as drivers of species co‐occurrence. Ecography, 41(8), 1233–1244. 10.1111/ecog.03148

[jane70187-bib-0014] Demby, A. H. , Inapogui, A. , Kargbo, K. , Koninga, J. , Kourouma, K. , Kanu, J. , Coulibaly, M. , Wagoner, K. D. , Ksiazek, T. G. , Peters, C. J. , Rollin, P. E. , & Bausch, D. G. (2001). Lassa fever in Guinea: II. Distribution and prevalence of Lassa virus infection in small mammals. Vector‐Borne and Zoonotic Diseases, 1(4), 283–297. 10.1089/15303660160025912 12653128

[jane70187-bib-0015] Denys, C. , Lecompte, E. , Calvet, E. , Camara, M. D. , Doré, A. , Koulémou, K. , Kourouma, F. , Soropogui, B. , Sylla, O. , Allali, B. K. , Kouassi‐Kan, S. , Akoua‐Koffi, C. , Ter Meulen, J. , & Koivogui, L. (2005). Community analysis of Muridae (Mammalia, Rodentia) diversity in Guinea: A special emphasis on Mastomys species and Lassa fever distributions. In B. A. Huber , B. J. Sinclair , & K.‐H. Lampe (Eds.), African biodiversity (pp. 339–350). Springer US. 10.1007/0-387-24320-8_34

[jane70187-bib-0016] Dijkstra, L. , Florczyk, A. J. , Freire, S. , Kemper, T. , Melchiorri, M. , Pesaresi, M. , & Schiavina, M. (2021). Applying the degree of urbanisation to the globe: A new harmonised definition reveals a different picture of global urbanisation. Journal of Urban Economics, 125(September), 103312. 10.1016/j.jue.2020.103312

[jane70187-bib-0017] Doser, J. W. , Finley, A. O. , Kéry, M. , & Zipkin, E. F. (2022). spOccupancy: An r package for single‐species, multi‐species, and integrated spatial occupancy models. Methods in Ecology and Evolution, 13, 1670–1678. 10.1111/2041-210X.13897

[jane70187-bib-0018] Ecke, F. , Han, B. A. , Hörnfeldt, B. , Khalil, H. , Magnusson, M. , Singh, N. J. , & Ostfeld, R. S. (2022). Population fluctuations and synanthropy explain transmission risk in rodent‐borne zoonoses. Nature Communications, 13(1), 7532. 10.1038/s41467-022-35273-7 PMC9729607 36477188

[jane70187-bib-0019] Eskew, E. A. , Bird, B. H. , Ghersi, B. M. , Bangura, J. , Basinski, A. J. , Amara, E. , Bah, M. A. , Kanu, M. C. , Kanu, O. T. , Lavalie, E. G. , Lungay, V. , Robert, W. , Vandi, M. A. , Fichet‐Calvet, E. , & Nuismer, S. L. (2024). Reservoir displacement by an invasive rodent reduces Lassa virus zoonotic spillover risk. Nature Communications, 15(1), 3589. 10.1038/s41467-024-47991-1 PMC11055883 38678025

[jane70187-bib-0020] Fichet‐Calvet, E. (2014). Chapter 5—Lassa fever: A rodent‐human interaction. In N. Johnson (Ed.), The role of animals in emerging viral diseases (pp. 89–123). Academic Press. 10.1016/B978-0-12-405191-1.00005-3

[jane70187-bib-0021] Fichet‐Calvet, E. , Becker‐Ziaja, B. , Koivogui, L. , & Gunther, S. (2014). Lassa serology in natural populations of rodents and horizontal transmission. Vector‐Borne and Zoonotic Diseases, 14(9), 665–674. 10.1089/vbz.2013.1484 25229705 PMC4170823

[jane70187-bib-0022] Fichet‐Calvet, E. , Koulemou, K. , Koivogui, L. , Soropogui, B. , Sylla, O. , Lecompte, E. , Daffis, S. , Bernard, A. K. , Kouassi, K. S. , Akoua‐Koffi, C. , & Denys, C. (2005). Spatial distribution of commensal rodents in regions with high and low Lassa fever prevalence in Guinea. Belgian Journal of Zoology, 135(December), 63–67. https://biblio.naturalsciences.be/associated_publications/bjz/bibliographic‐references/ISI_000203706900011

[jane70187-bib-0023] Fichet‐Calvet, E. , Lecompte, E. , Koivogui, L. , Soropogui, B. , Doré, A. , Kourouma, F. , Sylla, O. , Daffis, S. , Koulémou, K. , & Ter Meulen, J. (2007). Fluctuation of abundance and Lassa virus prevalence in *Mastomys natalensis* in Guinea, West Africa. Vector‐Borne and Zoonotic Diseases, 7(2), 119–128. 10.1089/vbz.2006.0520 17627428

[jane70187-bib-0024] Fichet‐Calvet, E. , Lecompte, E. , Veyrunes, F. , Barrière, P. , Nicolas, V. , & Koulémou, K. (2009). Diversity and dynamics in a community of small mammals in coastal Guinea, West Africa. Belgian Journal of Zoology, 139(2), 93–102. http://www.vliz.be/nl/open‐marien‐archief?module=ref&refid=204614

[jane70187-bib-0025] Fichet‐Calvet, E. , & Rogers, D. J. (2009). Risk maps of Lassa fever in West Africa. PLoS Neglected Tropical Diseases, 3(3), e388. 10.1371/journal.pntd.0000388 19255625 PMC2644764

[jane70187-bib-0026] Garba, M. , Dalecky, A. , Kadaoure, I. , Kane, M. , Hima, K. , Veran, S. , Gagare, S. , Gauthier, P. , Tatard, C. , Rossi, J.‐P. , & Dobigny, G. (2014). Spatial segregation between invasive and native commensal rodents in an urban environment: A case study in Niamey, Niger. PLoS One, 9(11), e110666. 10.1371/journal.pone.0110666 25379785 PMC4224371

[jane70187-bib-0027] Gibb, R. , Moses, L. M. , Redding, D. W. , & Jones, K. E. (2017). Understanding the cryptic nature of Lassa fever in West Africa. Pathogens and Global Health, 111(6), 276–288. 10.1080/20477724.2017.1369643 28875769 PMC5694855

[jane70187-bib-0028] Gibb, R. , Redding, D. W. , Chin, K. Q. , Donnelly, C. A. , Blackburn, T. M. , Newbold, T. , & Jones, K. E. (2020). Zoonotic host diversity increases in human‐dominated ecosystems. Nature, 584(7821), 398–402. 10.1038/s41586-020-2562-8 32759999

[jane70187-bib-0029] Gibb, R. , Redding, D. W. , Friant, S. , & Jones, K. E. (2025). Towards a ‘people and nature’ paradigm for biodiversity and infectious disease. Philosophical Transactions of the Royal Society, B: Biological Sciences, 380(1917), 20230259. 10.1098/rstb.2023.0259 PMC11712283 39780600

[jane70187-bib-0030] Glidden, C. K. , Nova, N. , Kain, M. P. , Lagerstrom, K. M. , Skinner, E. B. , Mandle, L. , Sokolow, S. H. , Plowright, R. K. , Dirzo, R. , de Leo, G. A. , & Mordecai, E. A. (2021). Human‐mediated impacts on biodiversity and the consequences for zoonotic disease spillover. Current Biology, 31(19), R1342–R1361. 10.1016/j.cub.2021.08.070 34637744 PMC9255562

[jane70187-bib-0031] Goyens, J. , Reijniers, J. , Borremans, B. , & Leirs, H. (2013). Density thresholds for Mopeia virus invasion and persistence in its host *Mastomys natalensis* . Journal of Theoretical Biology, 317(January), 55–61. 10.1016/j.jtbi.2012.09.039 23041432

[jane70187-bib-0032] Grant, D. S. , Engel, E. J. , Yerkes, N. R. , Kanneh, L. , Koninga, J. , Gbakie, M. A. , Alhasan, F. , Roberts Yerkes, N. , Kanneh, F. B. , Kanneh, I. M. , Kamara, F. K. , Momoh, M. , Yillah, M. S. , Foday, M. , Okoli, A. , Zeoli, A. , Weldon, C. , Bishop, C. M. , Zheng, C. , … Schieffelin, J. S. (2023). Seroprevalence of anti‐Lassa virus IgG antibodies in three districts of Sierra Leone: A cross‐sectional, population‐based study. PLoS Neglected Tropical Diseases, 17(2), e0010938. 10.1371/journal.pntd.0010938 36758101 PMC9946222

[jane70187-bib-0033] Halliday, F. W. , Rohr, J. R. , & Laine, A.‐L. (2020). Biodiversity loss underlies the dilution effect of biodiversity. Ecology Letters, 23(11), 1611–1622. 10.1111/ele.13590 32808427 PMC7693066

[jane70187-bib-0034] Han, B. A. , Schmidt, J. P. , Bowden, S. E. , & Drake, J. M. (2015). Rodent reservoirs of future zoonotic diseases. Proceedings of the National Academy of Sciences of the United States of America, 112(22), 7039–7044. 10.1073/pnas.1501598112 26038558 PMC4460448

[jane70187-bib-0035] Happi, A. N. , Olumade, T. J. , Ogunsanya, O. A. , Sijuwola, A. E. , Ogunleye, S. C. , Oguzie, J. U. , Nwofoke, C. , Ugwu, C. A. , Okoro, S. J. , Otuh, P. I. , Ngele, L. N. , Ojo, O. O. , Adelabu, A. , Adeleye, R. F. , Oyejide, N. E. , Njaka, C. S. , Heeney, J. L. , & Happi, C. T. (2022). Increased prevalence of Lassa fever virus‐positive rodents and diversity of infected species found during human Lassa fever epidemics in Nigeria. Microbiology Spectrum, 10(4), e0036622. 10.1128/spectrum.00366-22 35913205 PMC9430508

[jane70187-bib-0036] Hima, K. , Houémenou, G. , Badou, S. , Garba, M. , Dossou, H.‐J. , Etougbétché, J. , Gauthier, P. , Artige, E. , Fossati‐Gaschignard, O. , Gagaré, S. , Dobigny, G. , & Dalecky, A. (2019). Native and invasive small mammals in urban habitats along the commercial axis connecting Benin and Niger, West Africa. Diversity, 11(12), 238. 10.3390/d11120238

[jane70187-bib-0037] IPBES . (2020). Workshop Report on Biodiversity and Pandemics of the Intergovernmental Platform on Biodiversity and Ecosystem Services (IPBES). *Zenodo*, 10.5281/ZENODO.7432079

[jane70187-bib-0038] IUCN . (2016). The IUCN Red List of threatened species 2016: Mastomys natalensis. International Union for Conservation of Nature. 10.2305/IUCN.UK.2016-3.RLTS.T12868A22425266.en

[jane70187-bib-0039] Keenlyside, R. A. , McCormick, J. B. , Webb, P. A. , Smith, E. , Elliott, L. , & Johnson, K. M. (1983). Case‐control study of *Mastomys natalensis* and humans in Lassa virus‐infected households in Sierra Leone. The American Journal of Tropical Medicine and Hygiene, 32(4), 829–837. 6881432 10.4269/ajtmh.1983.32.829

[jane70187-bib-0040] Keesing, F. , & Ostfeld, R. S. (2021). Impacts of biodiversity and biodiversity loss on zoonotic diseases. Proceedings of the National Academy of Sciences of the United States of America, 118(17), e2023540118. 10.1073/pnas.2023540118 33820825 PMC8092607

[jane70187-bib-0041] Keesing, F. , & Ostfeld, R. S. (2024). Emerging patterns in rodent‐borne zoonotic diseases. Science, 385(6715), 1305–1310. 10.1126/science.adq7993 39298587

[jane70187-bib-0042] Kelly, J. D. , Bailor Barrie, M. , Ross, R. A. , Temple, B. A. , Moses, L. M. , & Bausch, D. G. (2013). Housing equity for health equity: A rights‐based approach to the control of Lassa fever in post‐war Sierra Leone. BMC International Health and Human Rights, 13(January), 2. 10.1186/1472-698X-13-2 23282054 PMC3562201

[jane70187-bib-0043] Kingdon, J. , & Happold, D. (2013). Mammals of Africa (Vol. 4). Bloomsbury Publishing.

[jane70187-bib-0044] Klitting, R. , Kafetzopoulou, L. E. , Thiery, W. , Dudas, G. , Gryseels, S. , Kotamarthi, A. , Vrancken, B. , Gangavarapu, K. , Momoh, M. , Sandi, J. D. , Goba, A. , Alhasan, F. , Grant, D. S. , Okogbenin, S. , Ogbaini‐Emovo, E. , Garry, R. F. , Smither, A. R. , Zeller, M. , Pauthner, M. G. , … Dellicour, S. (2022). Predicting the evolution of the Lassa virus endemic area and population at risk over the next decades. Nature Communications, 13(1), 5596. 10.1038/s41467-022-33112-3 PMC9515147 36167835

[jane70187-bib-0045] Leach, M. , Bett, B. , Said, M. , Bukachi, S. , Sang, R. , Anderson, N. , Machila, N. , Kuleszo, J. , Schaten, K. , Dzingirai, V. , Mangwanya, L. , Ntiamoa‐Baidu, Y. , Lawson, E. , Amponsah‐Mensah, K. , Moses, L. M. , Wilkinson, A. , Grant, D. S. , & Koninga, J. (2017). Local disease–ecosystem–livelihood dynamics: Reflections from comparative case studies in Africa. Philosophical Transactions of the Royal Society, B: Biological Sciences, 372(1725), 20160163. 10.1098/rstb.2016.0163 PMC5468688 28584171

[jane70187-bib-0046] Leirs, H. , Verheyen, W. , & Verhagen, R. (1996). Spatial patterns in *Mastomys natalensis* in Tanzania (Rodentia, Muridae). Mammalia, 60(4), 545–556. 10.1515/mamm.1996.60.4.545

[jane70187-bib-0047] Leirs, H. , Stenseth, N. C. , Nichols, J. D. , Hines, J. E. , Verhagen, R. , & Verheyen, W. (1997). Stochastic seasonality and nonlinear density‐dependent factors regulate population size in an African rodent. Nature, 389(6647), 176–180. 10.1038/38271 9296494

[jane70187-bib-0048] Leirs, H. , Verhagen, R. , & Verheyen, W. (1993). Productivity of different generations in a population of *Mastomys natalensis* rats in Tanzania. Oikos, 68(1), 53–60. 10.2307/3545308

[jane70187-bib-0049] Leirs, H. , Verhagen, R. , Verheyen, W. , Mwanjabe, P. , & Mbise, T. (1996). Forecasting rodent outbreaks in Africa: An ecological basis for mastomys control in Tanzania. Journal of Applied Ecology, 33(5), 937–943. 10.2307/2404675

[jane70187-bib-0050] Leski, T. A. , Stockelman, M. G. , Moses, L. M. , Park, M. , Stenger, D. A. , Ansumana, R. , Bausch, D. G. , & Lin, B. (2015). Sequence variability and geographic distribution of Lassa virus, Sierra Leone. Emerging Infectious Diseases, 21(4), 609–618. 10.3201/eid2104.141469 25811712 PMC4378485

[jane70187-bib-0051] Lippens, C. , Estoup, A. , Hima, M. K. , Loiseau, A. , Tatard, C. , Dalecky, A. , Ba, K. , Kane, M. , Diallo, M. , Sow, A. , Niang, Y. , Piry, S. , Berthier, K. , Leblois, R. , Duplantier, J.‐M. , & Brouat, C. (2017). Genetic structure and invasion history of the house mouse (*Mus musculus domesticus*) in Senegal, West Africa: A legacy of colonial and contemporary times. Heredity, 119(2), 64–75. 10.1038/hdy.2017.18 28353686 PMC5564374

[jane70187-bib-0052] Mantyka‐Pringle, C. S. , Visconti, P. , Di Marco, M. , Martin, T. G. , Rondinini, C. , & Rhodes, J. R. (2015). Climate change modifies risk of global biodiversity loss due to land‐cover change. Biological Conservation, 187(July), 103–111. 10.1016/j.biocon.2015.04.016

[jane70187-bib-0053] Mariën, J. , Kourouma, F. , Magassouba, N'F. , Leirs, H. , & Fichet‐Calvet, E. (2018). Movement patterns of small rodents in Lassa fever‐endemic villages in Guinea. EcoHealth, 15(2), 348–359. 10.1007/s10393-018-1331-8 29572697

[jane70187-bib-0054] Mariën, J. , Lo Iacono, G. , Rieger, T. , Magassouba, N. , Günther, S. , & Fichet‐Calvet, E. (2020). Households as hotspots of Lassa fever? Assessing the spatial distribution of Lassa virus‐infected rodents in rural villages of Guinea. Emerging Microbes & Infections, 9(1), 1055–1064. 10.1080/22221751.2020.1766381 32459576 PMC7336995

[jane70187-bib-0055] McCormick, J. B. , Webb, P. A. , Krebs, J. W. , Johnson, K. M. , & Smith, E. S. (1987). A prospective study of the epidemiology and ecology of Lassa fever. The Journal of Infectious Diseases, 155(3), 437–444. 10.1093/infdis/155.3.437 3805771

[jane70187-bib-0056] Mendoza, H. , Rubio, A. V. , García‐Peña, G. E. , Suzán, G. , & Simonetti, J. A. (2019). Does land‐use change increase the abundance of zoonotic reservoirs? Rodents say yes. European Journal of Wildlife Research, 66(1), 6. 10.1007/s10344-019-1344-9

[jane70187-bib-0057] Monadjem, A. , Taylor, P. J. , Denys, C. , & Cotterill, F. P. D. (2015). Rodents of sub‐Saharan Africa: A biogeographic and taxonomic synthesis. De Gruyter. 10.1515/9783110301915

[jane70187-bib-0058] Monath, T. P. , Newhouse, V. F. , Kemp, G. E. , Setzer, H. W. , & Cacciapuoti, A. (1974). Lassa virus isolation from *Mastomys natalensis* rodents during an epidemic in Sierra Leone. Science, 185(4147), 263–265. 10.1126/science.185.4147.263 4833828

[jane70187-bib-0059] Mulungu, L. S. , Ngowo, V. , Mdangi, M. , Katakweba, A. S. , Tesha, P. , Mrosso, F. P. , Mchomvu, M. , Sheyo, P. M. , & Kilonzo, B. S. (2013). Population dynamics and breeding patterns of multimammate mouse, *Mastomys natalensis* (Smith 1834), in irrigated rice fields in eastern Tanzania. Pest Management Science, 69(3), 371–377. 10.1002/ps.3346 22718470

[jane70187-bib-0060] Mylne, A. Q. N. , Pigott, D. M. , Longbottom, J. , Shearer, F. , Duda, K. A. , Messina, J. P. , Weiss, D. J. , Moyes, C. L. , Golding, N. , & Hay, S. I. (2015). Mapping the zoonotic niche of Lassa fever in Africa. Transactions of the Royal Society of Tropical Medicine and Hygiene, 109(8), 483–492. 10.1093/trstmh/trv047 26085474 PMC4501400

[jane70187-bib-0061] Naeem, S. , Duffy, J. E. , & Zavaleta, E. (2012). The functions of biological diversity in an age of extinction. Science, 336(6087), 1401–1406. 10.1126/science.1215855 22700920

[jane70187-bib-0062] Newbold, T. , Hudson, L. N. , Hill, S. L. L. , Contu, S. , Lysenko, I. , Senior, R. A. , Börger, L. , Bennett, D. J. , Choimes, A. , Collen, B. , Day, J. , de Palma, A. , Díaz, S. , Echeverria‐Londoño, S. , Edgar, M. J. , Feldman, A. , Garon, M. , Harrison, M. L. K. , Alhusseini, T. , … Purvis, A. (2015). Global effects of land use on local terrestrial biodiversity. Nature, 520(7545), 45–50. 10.1038/nature14324 25832402

[jane70187-bib-0063] Oksanen, J. , Simpson, G. L. , Blanchet, F. G. , Kindt, R. , Legendre, P. , Minchin, P. R. , & O'Hara, R. B. (2022). Vegan: Community ecology package . https://CRAN.R‐project.org/package=vegan

[jane70187-bib-0064] Olayemi, A. , Cadar, D. , Magassouba, N'F. , Obadare, A. , Kourouma, F. , Oyeyiola, A. , Fasogbon, S. , Igbokwe, J. , Rieger, T. , Bockholt, S. , Jérôme, H. , Schmidt‐Chanasit, J. , Garigliany, M. , Lorenzen, S. , Igbahenah, F. , Fichet, J.‐N. , Ortsega, D. , Omilabu, S. , Günther, S. , & Fichet‐Calvet, E. (2016). New hosts of the Lassa virus. Scientific Reports, 6(1), 25280. 10.1038/srep25280 27140942 PMC4853722

[jane70187-bib-0065] Olayemi, A. , Obadare, A. , Oyeyiola, A. , Fasogbon, S. , Igbokwe, J. , Igbahenah, F. , Ortsega, D. , Günther, S. , Verheyen, E. , & Fichet‐Calvet, E. (2018). Small mammal diversity and dynamics within Nigeria, with emphasis on reservoirs of the Lassa virus. Systematics and Biodiversity, 16(2), 118–127. 10.1080/14772000.2017.1358220

[jane70187-bib-0066] Olayemi, A. , Obadare, A. , Oyeyiola, A. , Igbokwe, J. , Fasogbon, A. , Igbahenah, F. , Ortsega, D. , Asogun, D. , Umeh, P. , Vakkai, I. , Abejegah, C. , Pahlman, M. , Becker‐Ziaja, B. , Günther, S. , & Fichet‐Calvet, E. (2016). Arenavirus diversity and phylogeography of *Mastomys natalensis* rodents, Nigeria. Emerging Infectious Diseases, 22(4), 694–697. 10.3201/eid2204.150155 26982388 PMC4806934

[jane70187-bib-0067] Olugasa, B. O. , Dogba, J. B. , Ogunro, B. , Odigie, E. A. , Nykoi, J. , Ojo, J. F. , Taiwo, O. , Kamara, A. , Mulbah, C. K. , & Fasunla, A. J. (2014). The rubber plantation environment and Lassa fever epidemics in Liberia, 2008–2012: A spatial regression. Spatial and Spatio‐temporal Epidemiology, 11, 163–174. 10.1016/j.sste.2014.04.005 25457605

[jane70187-bib-0068] Ostfeld, R. S. , & Holt, R. D. (2004). Are predators good for your health? Evaluating evidence for top‐down regulation of zoonotic disease reservoirs. Frontiers in Ecology and the Environment, 2(1), 13–20. 10.1890/1540-9295(2004)002[0013:APGFYH]2.0.CO;2

[jane70187-bib-0069] Parsons, A. W. , Clark, J. S. , & Kays, R. (2022). Monitoring small mammal abundance using NEON data: Are calibrated indices useful? Journal of Mammalogy, 104, 292–302. 10.1093/jmammal/gyac096

[jane70187-bib-0070] Pebesma, E. (2018). Simple features for r: Standardized support for spatial vector data. The R Journal, 10(1), 439–446. https://journal.r‐project.org/archive/2018/RJ‐2018‐009/index.html

[jane70187-bib-0071] Pei, S. , Yu, P. , Raghwani, J. , Wang, Y. , Liu, Z. , Li, Y. , Cheng, Y. , Lin, Q. , Song, C. , Dharmarajan, G. , Faust, C. L. , Tian, Y. , Xu, Y. , Liang, Y. , Qu, J. , Wei, J. , Li, S. , Zhang, T. , Ma, C. , … Tian, H. (2024). Anthropogenic land consolidation intensifies zoonotic host diversity loss and disease transmission in human habitats. Nature Ecology & Evolution, 9(1), 99–110. 10.1038/s41559-024-02570-x 39558089

[jane70187-bib-0072] Polis, G. A. , & Holt, R. D. (1992). Intraguild predation: The dynamics of complex trophic interactions. Trends in Ecology & Evolution, 7(5), 151–154. 10.1016/0169-5347(92)90208-S 21235990

[jane70187-bib-0073] QIAGEN . (2023). DNeasy blood & tissue kits . https://www.qiagen.com/us/products/discovery‐and‐translational‐research/dna‐rna‐purification/dna‐purification/genomic‐dna/dneasy‐blood‐and‐tissue‐kit

[jane70187-bib-0074] R Core Team . (2021). R: A language and environment for statistical computing. R Foundation for Statistical Computing. https://www.R‐project.org/

[jane70187-bib-0075] Redding, D. W. , Gibb, R. , Dan‐Nwafor, C. C. , Ilori, E. A. , Yashe, R. U. , Oladele, S. H. , Amedu, M. O. , Iniobong, A. , Attfield, L. A. , Donnelly, C. A. , Abubakar, I. , Jones, K. E. , & Ihekweazu, C. (2021). Geographical drivers and climate‐linked dynamics of Lassa fever in Nigeria. Nature Communications, 12(1), 5759. 10.1038/s41467-021-25910-y PMC8486829 34599162

[jane70187-bib-0076] Redding, D. W. , Moses, L. M. , Cunningham, A. A. , Wood, J. , & Jones, K. E. (2016). Environmental‐mechanistic modelling of the impact of global change on human zoonotic disease emergence: A case study of Lassa fever. Methods in Ecology and Evolution, 7(6), 646–655. 10.1111/2041-210X.12549

[jane70187-bib-0077] Sala, O. E. , Chapin, F. S., III , Armesto, J. J. , Berlow, E. , Bloomfield, J. , Dirzo, R. , Stuart Chapin, F. , Huber‐Sanwald, E. , Huenneke, L. F. , Jackson, R. B. , Kinzig, A. , Leemans, R. , Lodge, D. M. , Mooney, H. A. , Oesterheld, Martín , Poff, N. L. R. , Sykes, M. T. , Walker, B. H. , Walker, M. , & Wall, D. H. (2000). Global biodiversity scenarios for the year 2100. Science, 287(5459), 1770–1774. 10.1126/science.287.5459.1770 10710299

[jane70187-bib-0078] Salkeld, D. J. , Padgett, K. A. , & Jones, J. H. (2013). A meta‐analysis suggesting that the relationship between biodiversity and risk of zoonotic pathogen transmission is idiosyncratic. Ecology Letters, 16(5), 679–686. 10.1111/ele.12101 23489376 PMC7163739

[jane70187-bib-0079] Saluzzo, J. F. , Adam, F. , McCormick, J. B. , & Digoutte, J. P. (1988). Lassa fever virus in Senegal. Journal of Infectious Diseases, 157(3), 605. https://www.ncbi.nlm.nih.gov/pubmed/3343533 3343533 10.1093/infdis/157.3.605

[jane70187-bib-0080] Shannon, C. E. (1948). A mathematical theory of communication. Bell System Technical Journal, 27(3), 379–423. 10.1002/j.1538-7305.1948.tb01338.x

[jane70187-bib-0081] Simons, D. (2022). Lassa fever cases suffer from severe underreporting based on reported fatalities. International Health, 15, ihac076. 10.1093/inthealth/ihac076 PMC10472868 36413115

[jane70187-bib-0082] Simons, D. , Attfield, L. A. , Jones, K. E. , Watson‐Jones, D. , & Kock, R. (2023). Rodent trapping studies as an overlooked information source for understanding endemic and novel zoonotic spillover. PLoS Neglected Tropical Diseases, 17(1), e0010772. 10.1371/journal.pntd.0010772 36689474 PMC9894545

[jane70187-bib-0083] Simons, D. , Rivero, R. , Martinez‐Checa Guiote, A. , Gordon, H. L. M. , Milne, G. C. , Rickard, G. , Redding, D. W. , & Seifert, S. N. (2025). Protocol to produce a systematic arenavirus and hantavirus host‐pathogen database: Project ArHa. Wellcome Open Research, 10, 227. 10.12688/wellcomeopenres.24037.2 40927039 PMC12415512

[jane70187-bib-0084] Stryjek, R. , Kalinowski, A. , & Parsons, M. H. (2019). Unbiased sampling for rodents and other small mammals: How to overcome neophobia through use of an electronic‐triggered live trap—A preliminary test. Frontiers in Ecology and Evolution, 7, 11. 10.3389/fevo.2019.00011

[jane70187-bib-0085] Taylor, K. D. , Hammond, L. E. , & Quy, R. J. (1974). The reactions of common rats to four types of live‐capture trap. The Journal of Applied Ecology, 11(2), 453. 10.2307/2402199

[jane70187-bib-0086] Vrbanec, L. , Matijević, V. , & Guenther, A. (2021). Enhanced problem‐solving ability as an adaptation to urban environments in house mice. Proceedings of the Royal Society B: Biological Sciences, 288(1945), 20202504. 10.1098/rspb.2020.2504 PMC7934975 33593181

[jane70187-bib-0087] Watanabe, S. (2010). Asymptotic equivalence of Bayes cross validation and widely applicable information criterion in singular learning theory. Journal of Machine Learning Research, 11(December), 3571–3594. 10.5555/1756006.1953045

[jane70187-bib-0088] World Health Organisation . (2022). Lassa fever . https://www.who.int/health‐topics/lassa‐fever#tab=tab_1

[jane70187-bib-0089] WorldPop . (2025). Open spatial demographic data and research . https://hub.worldpop.org/

[jane70187-bib-0090] Wulff, H. , Fabiyi, A. , & Monath, T. P. (1975). Recent isolations of Lassa virus from Nigerian rodents. Bulletin of the World Health Organization, 52(4–6), 609–613. 1085216 PMC2366652

[jane70187-bib-0091] Yadouleton, A. , Agolinou, A. , Kourouma, F. , Saizonou, R. , Pahlmann, M. , Bedie, S. K. , Bankole, H. , Becker‐Ziaja, B. , Gbaguidi, F. , Thielebein, A. , Magassouba, N'F. , Duraffour, S. , Baptiste, J.‐P. , Günther, S. , & Fichet‐Calvet, E. (2019). Lassa virus in pygmy mice, Benin, 2016–2017. Emerging Infectious Diseases, 25(10), 1977–1979. 10.3201/eid2510.180523 31365854 PMC6759236

[jane70187-bib-0092] Young, H. S. , Dirzo, R. , Helgen, K. M. , McCauley, D. J. , Billeter, S. A. , Kosoy, M. Y. , Osikowicz, L. M. , Salkeld, D. J. , Young, T. P. , & Dittmar, K. (2014). Declines in large wildlife increase landscape‐level prevalence of rodent‐borne disease in Africa. Proceedings of the National Academy of Sciences of the United States of America, 111(19), 7036–7041. 10.1073/pnas.1404958111 24778215 PMC4024866

[jane70187-bib-0093] Young, H. S. , McCauley, D. J. , Dirzo, R. , Nunn, C. L. , Campana, M. G. , Agwanda, B. , Otarola‐Castillo, E. R. , Castillo, E. R. , Pringle, R. M. , Veblen, K. E. , Salkeld, D. J. , Stewardson, K. , Fleischer, R. , Lambin, E. F. , Palmer, T. M. , & Helgen, K. M. (2017). Interacting effects of land use and climate on rodent‐borne pathogens in Central Kenya. Philosophical Transactions of the Royal Society, B: Biological Sciences, 372(1722), 20160116. 10.1098/rstb.2016.0116 PMC5413868 28438909

