# Supplementary material for: Land use gradients drive spatial variation in Lassa fever host communities in the Eastern Province of Sierra Leone
Source: J Anim Ecol. 2025 Nov 21;95(2):296–312. doi: 10.1111/1365-2656.70187 (PMC12868399; doi:10.1111/1365-2656.70187)
Supplement: Supplementary file 1 — Table S1: Model comparison for the Bayesian spatial latent factor multi‐species occupancy model. Models are ordered by WAIC. ELPD, expected log pointwise predictive density, pD, the effective number of parameters, WAIC, widely applicable information criterion. Table S2: Sampling of rodents for LASV. Figure S1: Trap locations and number of trap nights in (A) Baiama, (B) Lalehun, (C) Lambayama and (D) Seilama. Each cell represents a 49 m2 area. The colour of cells relates to the trapping effort within that area in number of trap nights. The facets relate to the land use type of the trapping site. Images obtained from Google maps, copyright 2023 CNES/Airbus, Maxar Technologies. Figure S2: Schematic diagram of conversion from trap locations to grids. (A) Individual traps were placed in a grid structure in a pre‐specified location. While attempts were made to keep trap locations over repeated visits individual traps were often placed in slightly different locations. This is shown in the schematic using different colours to represent different visits. (B) To harmonise the locations of traps to coordinates that could be used in the spatial occupancy model we aligned a regular grid with grid cell sizes of 49 m2 over the trapping area and assigned individuals traps to these cells. (C) The number of traps and therefore the number of trapnights within each grid cell was aggregated for each visit. The number of trap nights informed the detection component of the species occupancy model. Detection histories were produced for each grid cell that was sampled for each species. Grid cells were assigned a 1 if any trap within the grid cell detected the species and 0 otherwise. Figure S3: (A) Species accumulation curves by village site. (B) Species accumulation curves by village site stratified by land use type. The x‐axis represents the number of unique 49 m2 grid cells (sites) accumulated, not individual trap nights. Figure S4: Conceptual model used to identify potential causal path [file JANE-95-296-s001.zip › jane70187-sup-0003-TableS1-S2-FigureS1-S8@Supporting_Information.docx]

# Supporting Information

## Supporting Information 1

**Supporting_Information_1.pdf – Study protocols**

## Supporting Information 2

**Supporting_Information_2.pdf – Taxonomic key**

## Supplementary Table 1

*Supplementary Table 1: Model comparison for the Bayesian spatial latent factor multi-species occupancy model. Models are ordered by WAIC. ELPD - Expected log pointwise predictive density, pD - The effective number of parameters, WAIC - Widely Applicable Information Criterion.*

| Model | Occupancy component formula | ELPD | pD | WAIC |
| --- | --- | --- | --- | --- |
| 2 | ~landuse + village | -2,209.733 | 117.92741 | 4,655.320 |
| 8 | ~landuse + village + scale(distance_building) | -2,209.817 | 119.22588 | 4,658.086 |
| 11^*^ | ~landuse + village + scale(distance_building) | -2,209.822 | 119.78854 | 4,659.221 |
| 10^*^ | ~landuse + village | -2,213.714 | 118.20917 | 4,663.845 |
| 5 | ~group_landuse + scale(elevation) | -2,201.773 | 130.40165 | 4,664.349 |
| 9 | ~landuse + village + scale(elevation) | -2,211.811 | 126.29186 | 4,676.205 |
| 7 | ~landuse + village + scale(distance_building) + scale(elevation) | -2,224.290 | 115.26248 | 4,679.105 |
| 1 | ~landuse | -2,250.034 | 89.93717 | 4,679.943 |
| 4 | ~group_landuse + scale(distance_building) | -2,225.106 | 121.20351 | 4,692.618 |
| 3 | ~group_landuse | -2,230.146 | 119.44685 | 4,699.186 |
| 6 | ~group_landuse + scale(distance_building) + scale(elevation) | -2,228.705 | 127.81074 | 4,713.032 |
| 0 | intercept only | -2,307.128 | 77.91029 | 4,770.077 |
| ^*^These models are versions of Models 2 and 8, respectively, run with longer MCMC chains as a sensitivity analysis to confirm model convergence and stability of the WAIC rankings. They were not part of the primary model selection set. | | | | |

## Supplementary Table 2

**lasv_detections.csv – Sampling of rodents for LASV**

## Supplementary Figure 1A-D

**
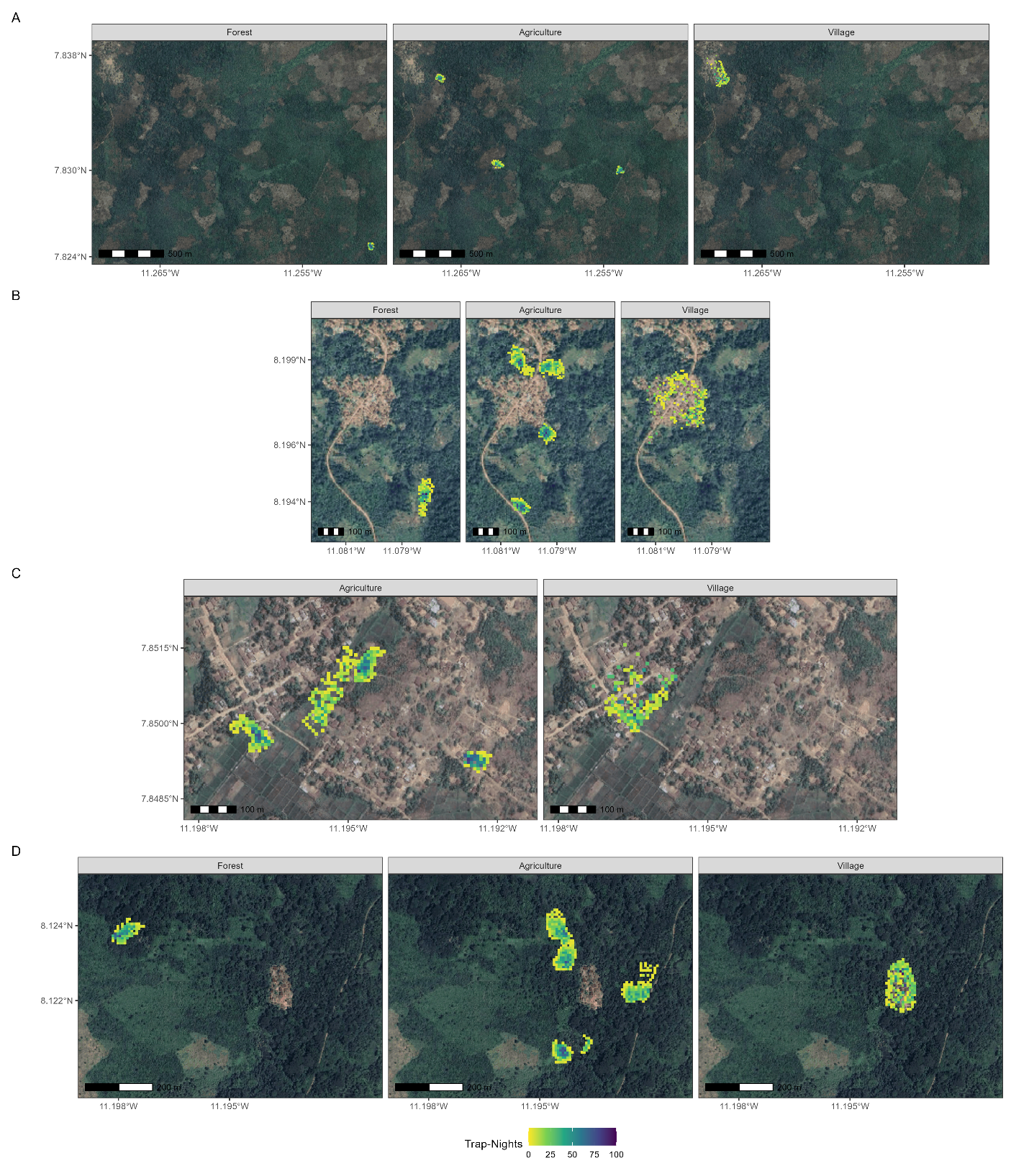
**

*Supplementary Figure 1. Trap locations and number of trap nights in A) Baiama, B) Lalehun, C) Lambayama and D) Seilama. Each cell represents a 49m^2^ area. The colour of cells relates to the trapping effort within that area in number of trap nights. The facets relate to the land use type of the trapping site. Images obtained from Google maps, copyright 2023 CNES/Airbus, Maxar Technologies.*

## Supplementary Figure 2


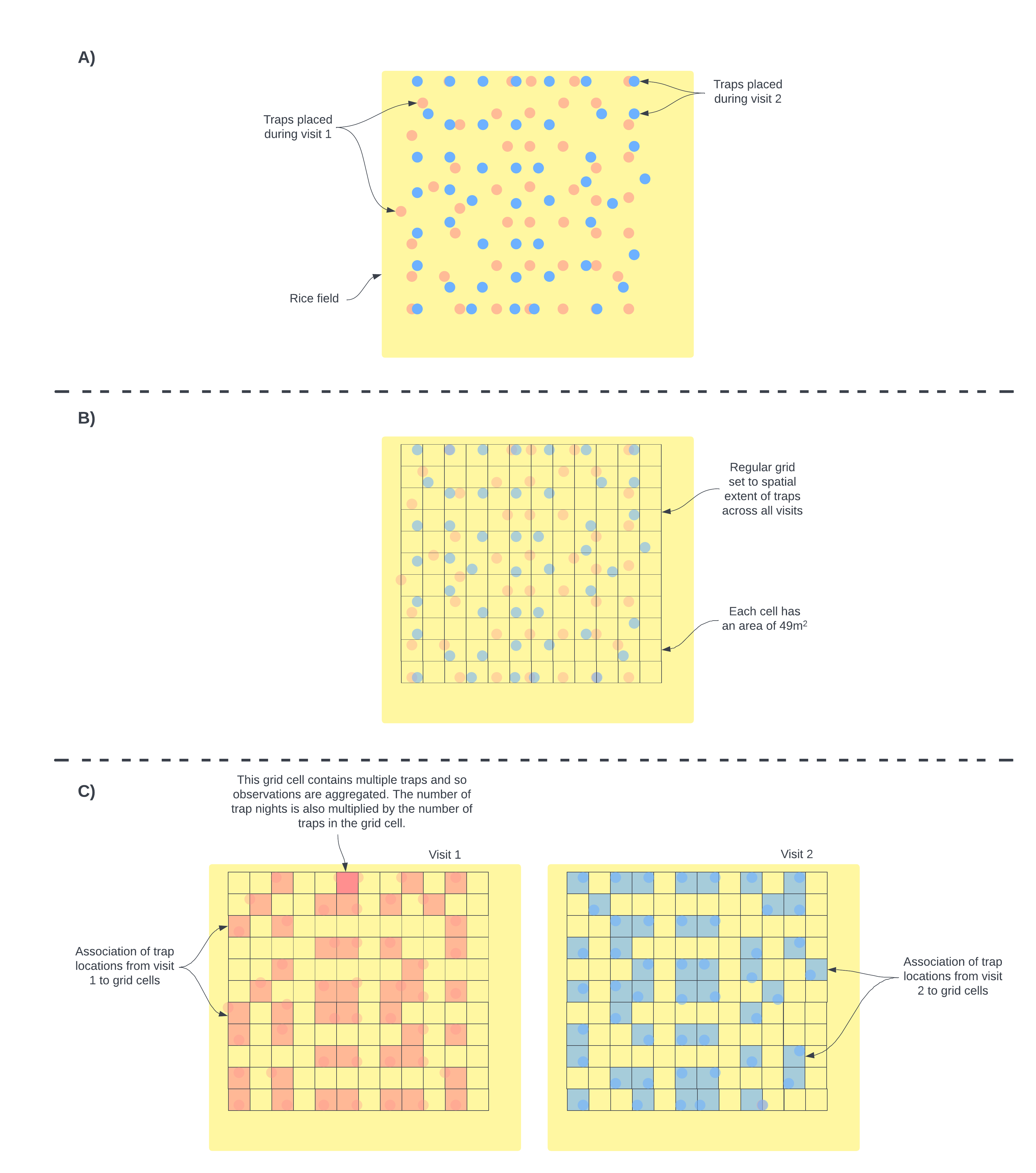


Supplementary Figure 2. Schematic diagram of conversion from trap locations to grids. A) Individual traps were placed in a grid structure in a pre-specified location. While attempts were made to keep trap locations over repeated visits individual traps were often placed in slightly different locations. This is shown in the schematic using different colours to represent different visits. B) To harmonize the locations of traps to coordinates that could be used in the spatial occupancy model we aligned a regular grid with grid cell sizes of 49m^2^ over the trapping area and assigned individuals traps to these cells. C) The number of traps and therefore the number of trapnights within each grid cell was aggregated for each visit. The number of trap nights informed the detection component of the species occupancy model. Detection histories were produced for each grid cell that was sampled for each species. Grid cells were assigned a 1 if any trap within the grid cell detected the species and 0 otherwise.

## Supplementary Figure 3


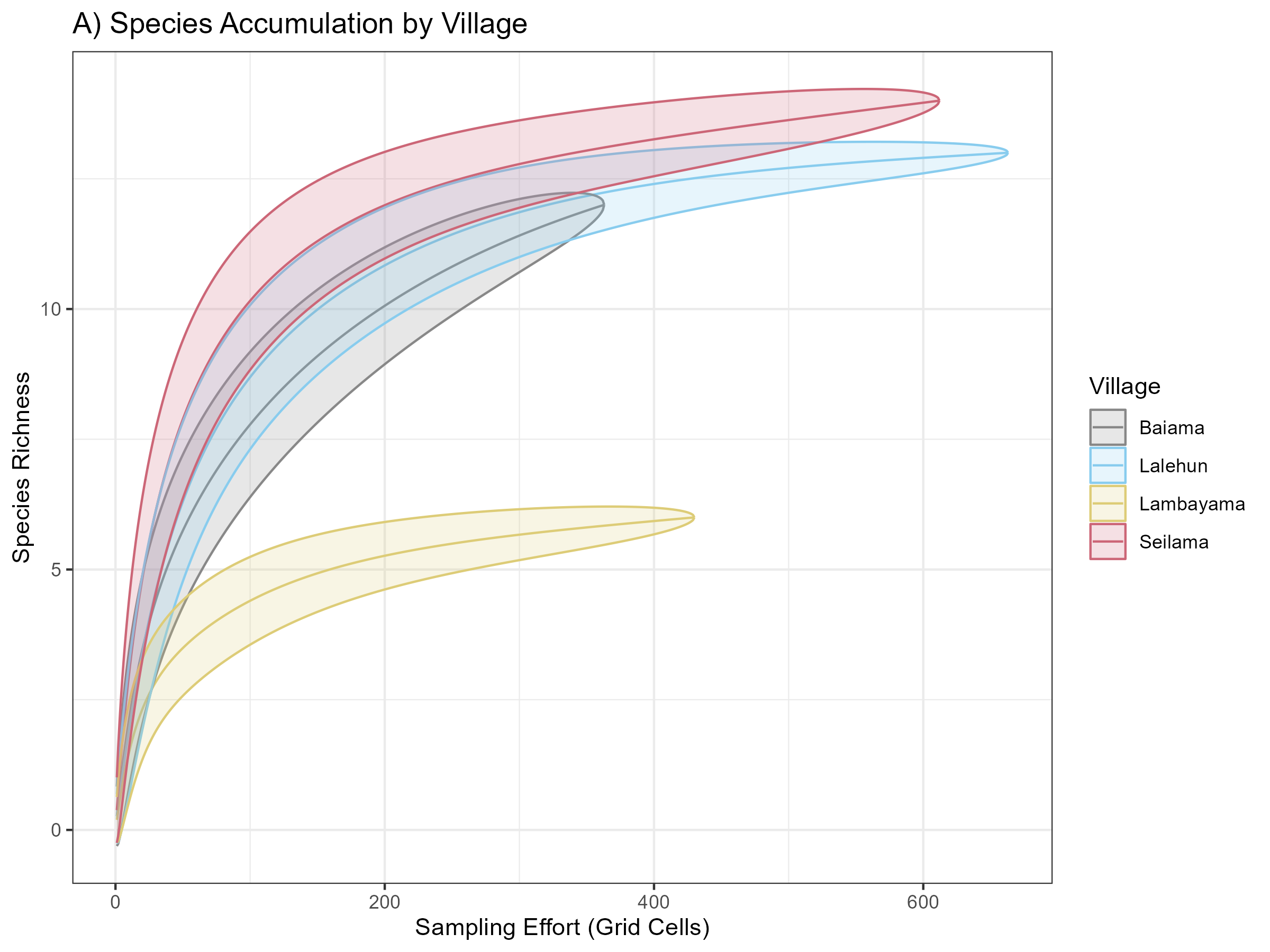

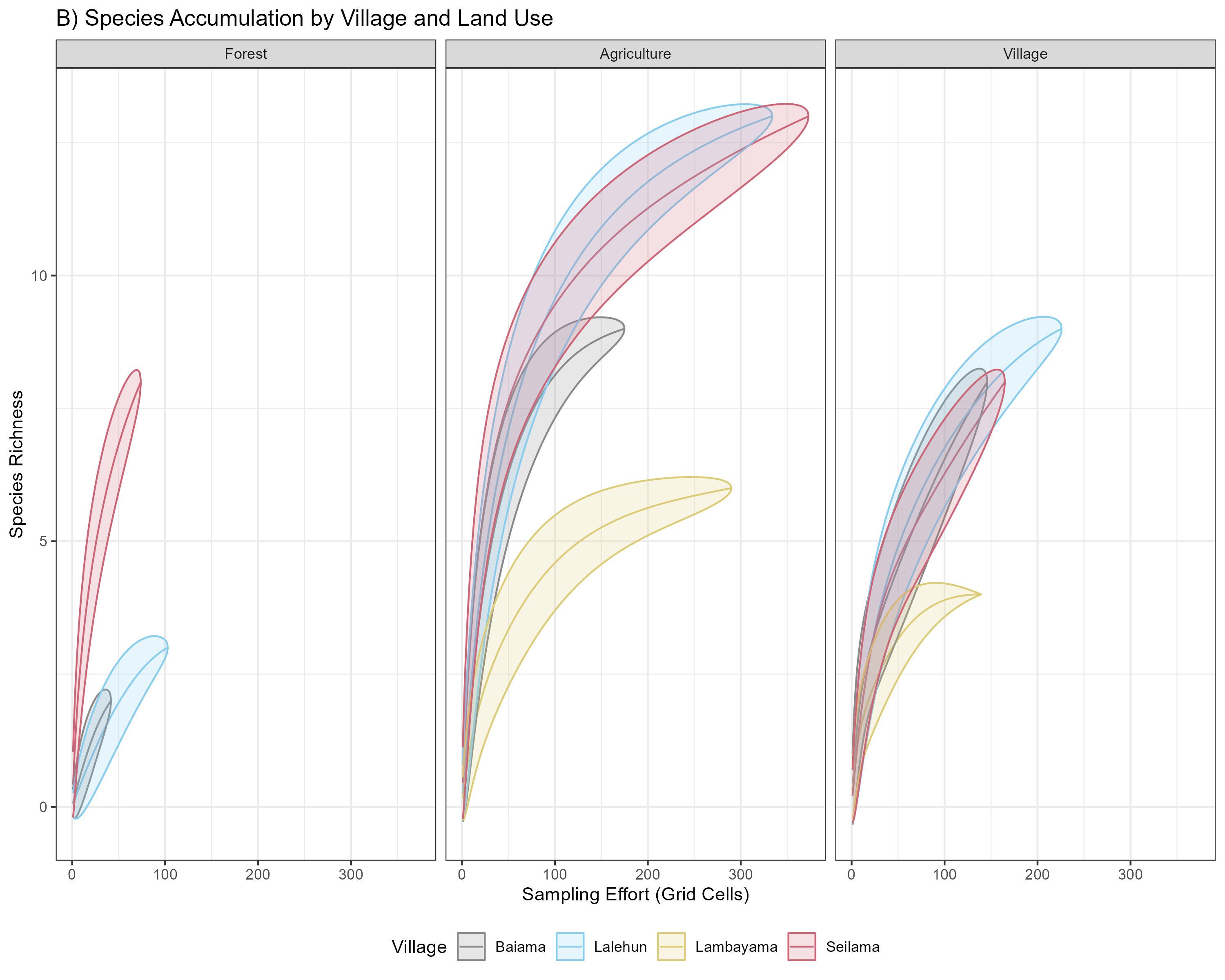
 *Supplementary Figure 3. A) Species accumulation curves by village site. B) Species accumulation curves by village site stratified by land use type. The x-axis represents the number of unique 49 m² grid cells (sites) accumulated, not individual trap-nights.*

## Supplementary Figure 4


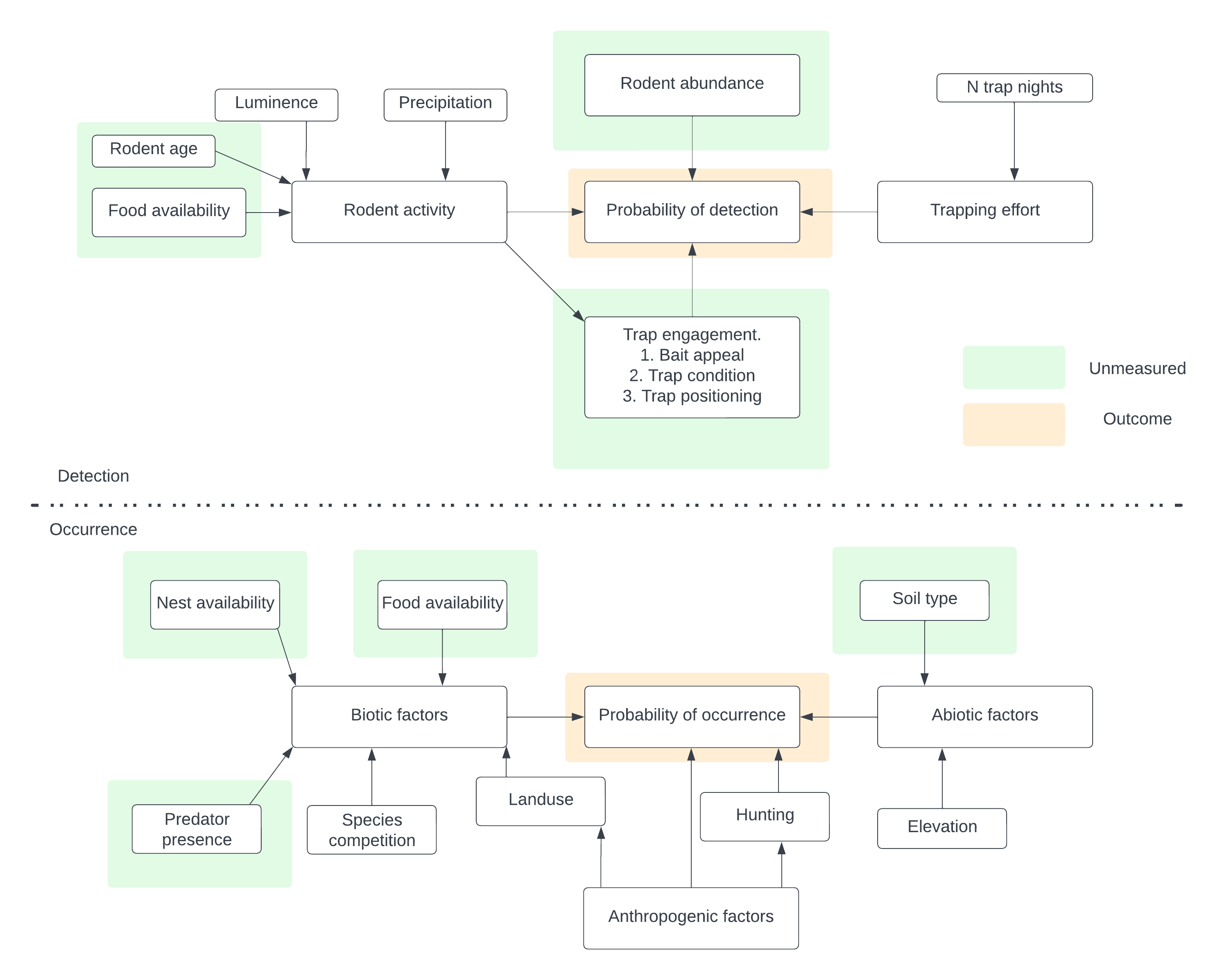


Supplementary Figure 4. Conceptual model used to identify potential causal pathways for inclusion of variables for the occupancy and detection model specification.

## Supplementary Figure 5


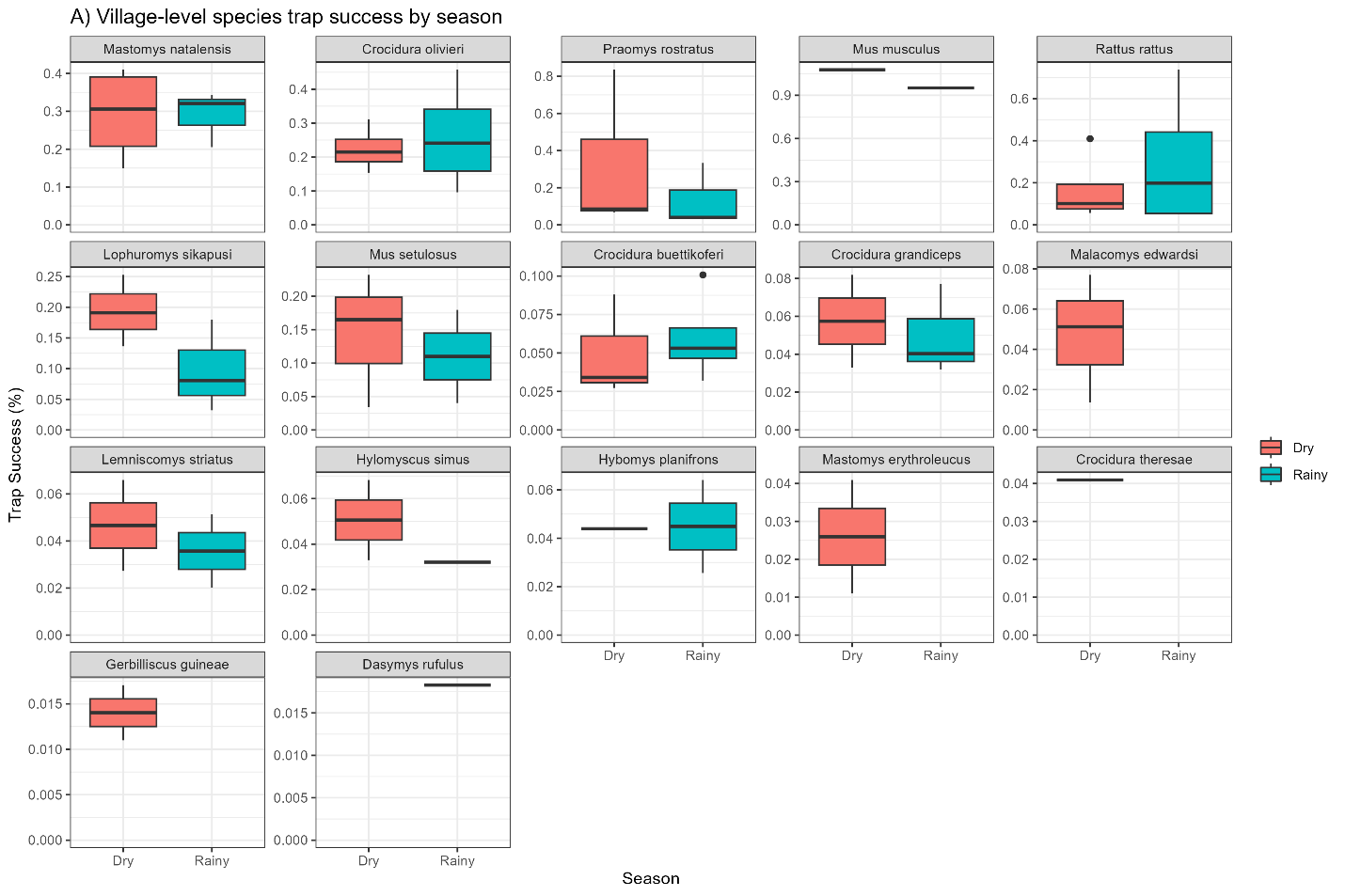


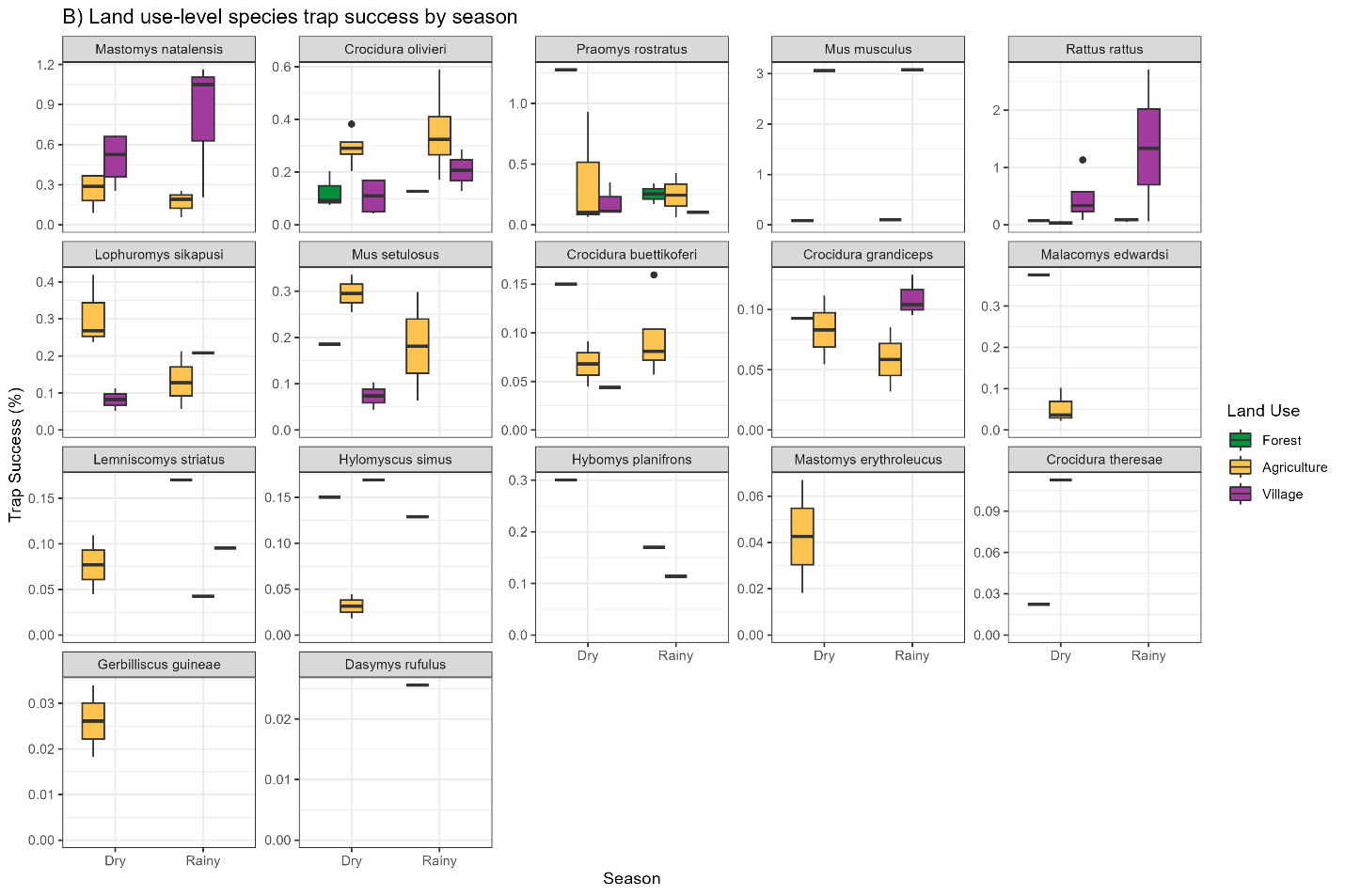
*Supplementary material 5. A) Trap success for all species by season (Rainy = May-October, Dry = November-April) of trapping activity. B) Trap success for all species by season (Rainy = May-October, Dry = November-April) stratified by land use type.*

## Supplementary Figure 6


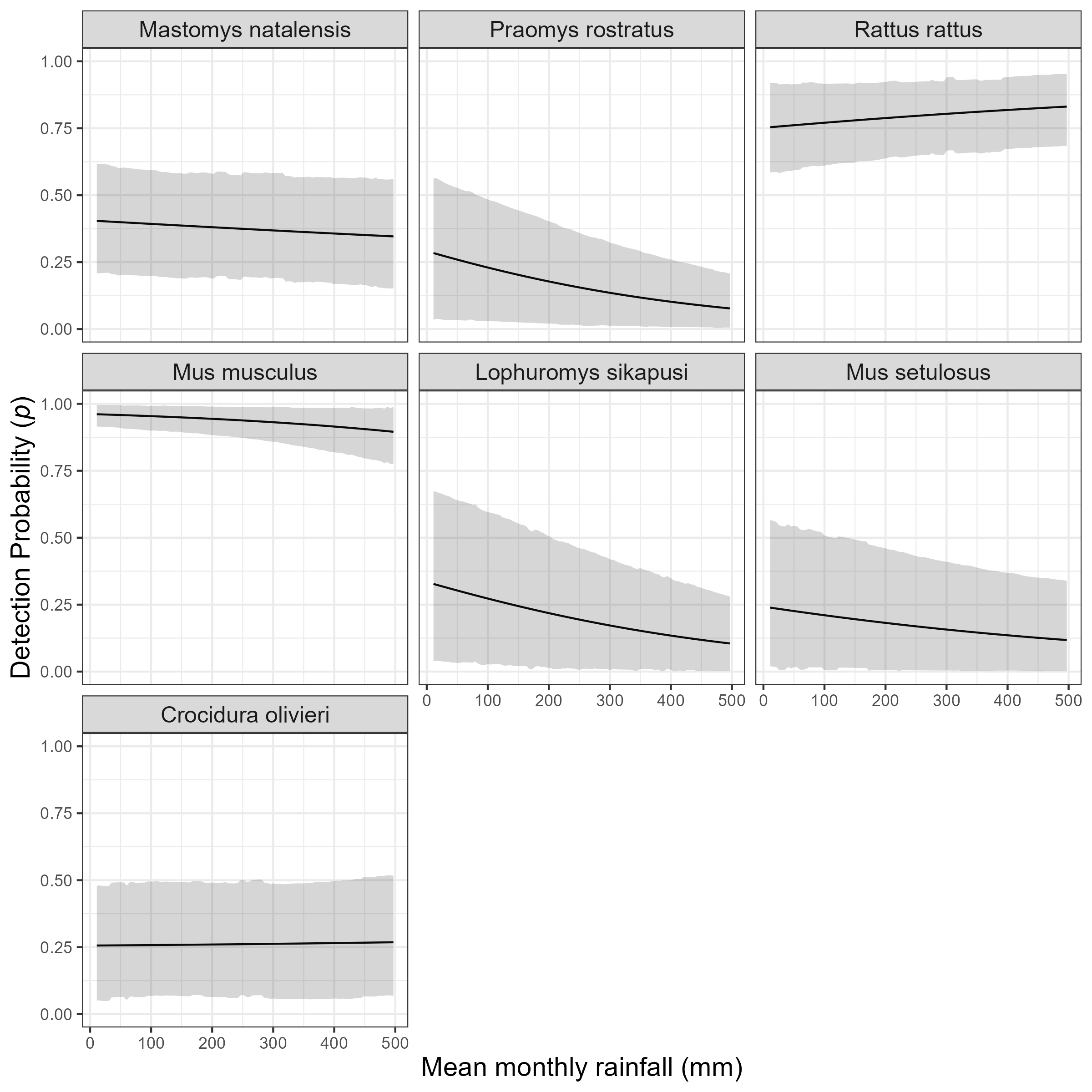


Supplementary Figure 6: The marginal effect of mean monthly rainfall on the probability of detection of a species in a grid cell. The black line shows the mean modelled probability of detection for the amount of monthly rainfall, the shaded grey region represents the 95% Credible Interval (CrI). Probability of detection varies by species with higher values for the invasive rodent species M. musculus and R. rattus, than the native rodent species. There is a general response of decreasing probability of detection with increasing rainfall.

## Supplementary Figure 7


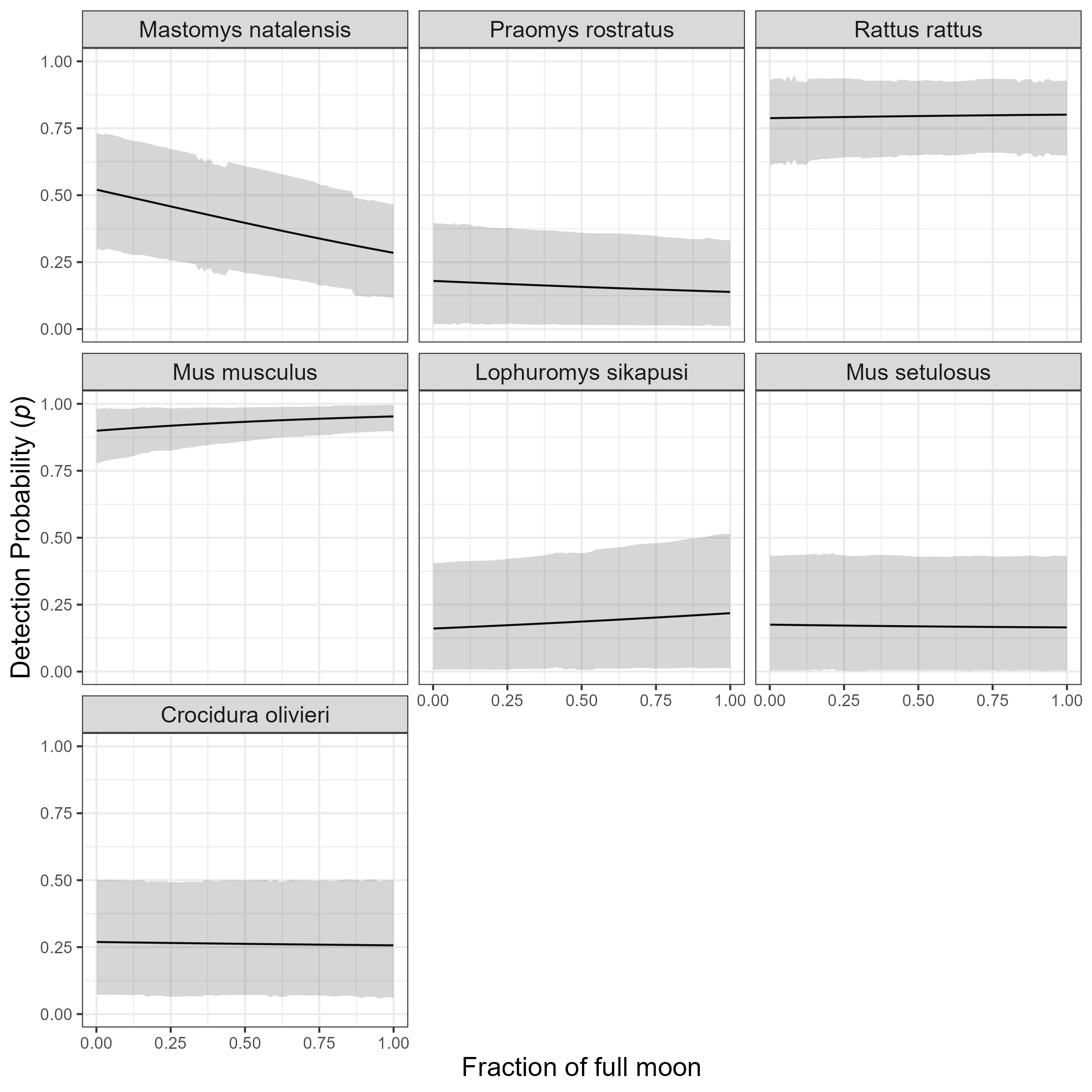


Supplementary Figure 7: The marginal effect of the fraction of the full moon on the probability of detection of a species in a grid cell. The black line shows the mean modelled probability of detection for the moon phase, the shaded grey region represents the 95% CrI. Probability of detection varies by species with higher values for the invasive rodent species M. musculus and R. rattus, than the native rodent species. There is no important response to moon phase for most species. The probability of detection appears to fall for M. natalensis with increasing moon phase but the credible intervals overlap for the entire range.

## Supplementary Figure 8


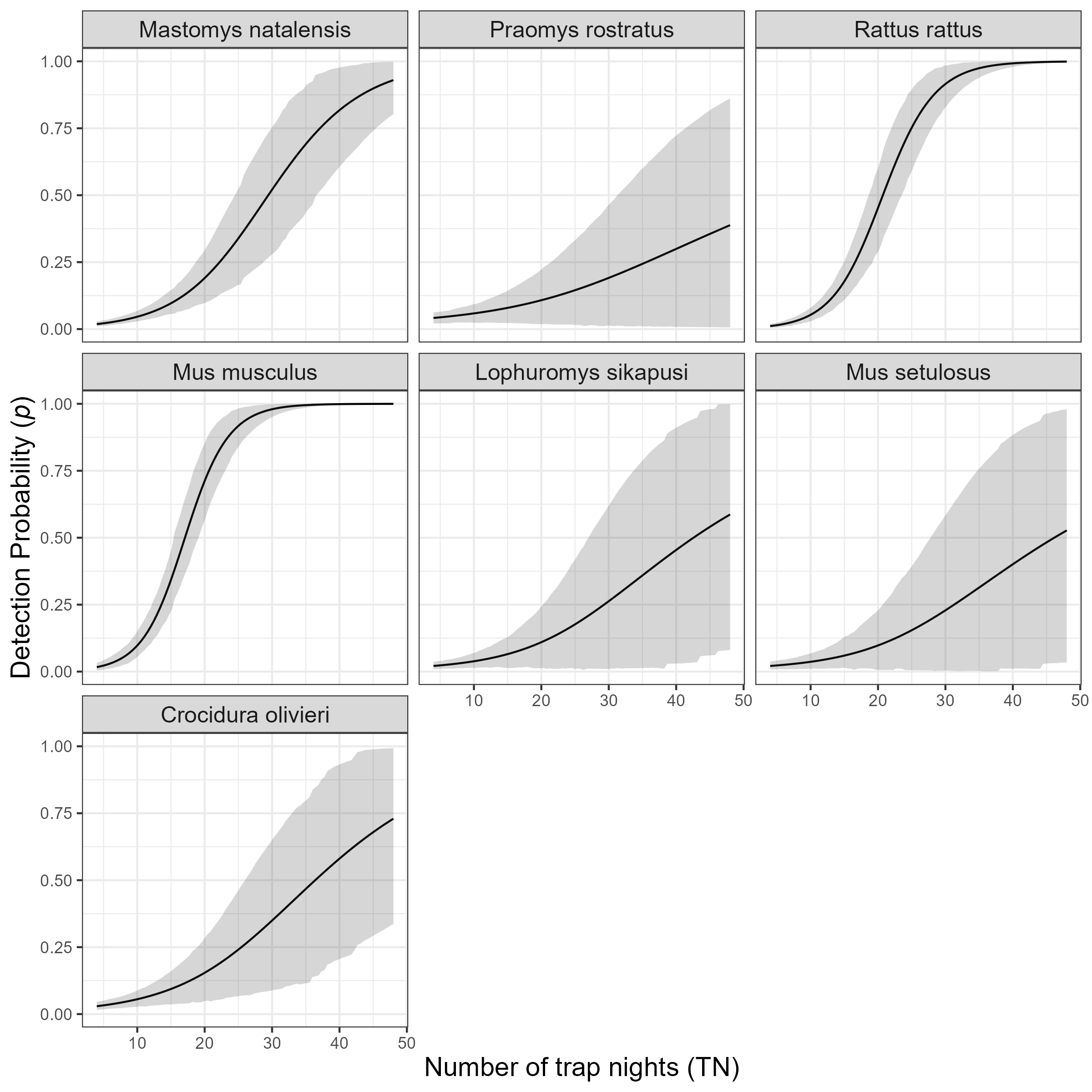


Supplementary Figure 8: The marginal effect of trapping effort (TN) on the probability of detection of a species in a grid cell. The black line shows the mean modelled probability of detection trapping effort, the shaded grey region represents the 95% CrI. Probability of detection is low for all species at low levels of TN. The probability of detection with increasing TN varies by species. The invasive rodent species M. musculus and R. rattus show a sinusoidal response over the range of TNs that were observed in this study with the probability of detection being greater than 50% at relatively low numbers of TN (17 and 22 respectively). A much greater trapping effort were required to obtain the same probability of detection for native rodent species. Only M. natalensis reached 50% detection among the range of TN conducted within grid cells in this study, requiring 29 TN to reach a probability of 50% detection.
