# Supplementary material for: Land use gradients drive spatial variation in Lassa fever host communities in the Eastern Province of Sierra Leone
Source: J Anim Ecol. 2025 Nov 21;95(2):296–312. doi: 10.1111/1365-2656.70187 (PMC12868399; doi:10.1111/1365-2656.70187)
Supplement: Supplementary file 1 — Table S1: Model comparison for the Bayesian spatial latent factor multi‐species occupancy model. Models are ordered by WAIC. ELPD, expected log pointwise predictive density, pD, the effective number of parameters, WAIC, widely applicable information criterion. Table S2: Sampling of rodents for LASV. Figure S1: Trap locations and number of trap nights in (A) Baiama, (B) Lalehun, (C) Lambayama and (D) Seilama. Each cell represents a 49 m2 area. The colour of cells relates to the trapping effort within that area in number of trap nights. The facets relate to the land use type of the trapping site. Images obtained from Google maps, copyright 2023 CNES/Airbus, Maxar Technologies. Figure S2: Schematic diagram of conversion from trap locations to grids. (A) Individual traps were placed in a grid structure in a pre‐specified location. While attempts were made to keep trap locations over repeated visits individual traps were often placed in slightly different locations. This is shown in the schematic using different colours to represent different visits. (B) To harmonise the locations of traps to coordinates that could be used in the spatial occupancy model we aligned a regular grid with grid cell sizes of 49 m2 over the trapping area and assigned individuals traps to these cells. (C) The number of traps and therefore the number of trapnights within each grid cell was aggregated for each visit. The number of trap nights informed the detection component of the species occupancy model. Detection histories were produced for each grid cell that was sampled for each species. Grid cells were assigned a 1 if any trap within the grid cell detected the species and 0 otherwise. Figure S3: (A) Species accumulation curves by village site. (B) Species accumulation curves by village site stratified by land use type. The x‐axis represents the number of unique 49 m2 grid cells (sites) accumulated, not individual trap nights. Figure S4: Conceptual model used to identify potential causal path [file JANE-95-296-s001.zip › jane70187-sup-0005-Supinfo2@Supporting_Information_2.pdf]

Small mammal identification key – November 2020

Morphological data for rodent species contained in tables obtained from: Monadjem, Ara, Peter Taylor, and Christiane Denys. *Rodents of Sub-Saharan Africa: A Biogeographic and Taxonomic Synthesis*. Walter de Gruyter, 2015.
Morphological data for shrew species contained in tables obtained from: Kingdon, Jonathan, and David Happold. 'Mammals of Africa' 4 (2013).

HB = head and body
GLS = greatest length of skull
HF = hind foot

Squirrels

Epixerus ebii (Temminck, 1853) – Western palm squirrel

Generally large, squirrel-shaped. Generally dark brown pelage. Long ears. 5 digits hindfoot, 4 forefoot. 4 pairs of nipples. Feeds on hard shelled nuts and fruit. Primarily active in the morning.
<epixerus ebii image>

Table 1: Data obtained from Monadgem et al. 2015

	Mean (mm)	Range (mm)
Mass	470.3	346-543
HB	263.8	240-280
Tail	283.8	260-320
HF	65.1	62.3-68.3
Ear	19.4	18-20
GLS	68.4	64.9-70.5

Funisciurus pyrropus (F.Cuvier, 1833) – Fire footed rope squirrel

Smaller than most other squirrels (100-300g). Short legs and long tail. Moderate sized ears and rounded. 2 pairs of nipples.
<funisciurus pyrropus image>
Carcass and upper cheek teeth, note the small premolar. Typically small home range 1-5 ha. Frugivorous diet. Identified by presence of single pale flank stripe, without conspicuous black border stripes, which may or may not be broken into spots and with or without a single dark border stripe above or below but not both. Bright orange or red on the side of the face, the limbs and a conspicuous white flank stripe.

Table 2: Data obtained from Monadgem et al. 2015

	Mean (mm)	Range (mm)
Mass	241.6	187-289
HB	198.8	183-215
Tail	178	154-210
HF	46.2	41-52
Ear	17.2	11.7-20
GLS	49	46.7-51.9

Heliosciurus

3 species believed endemic in SL. Typically 150-390g, long legs and tail. Relatively small ears and rounded. The tail is always longer than the body length and very bushy. 3 pairs of nipples. Primarily frugivorous.

Heliosciurus gambianus (Ogilby, 1835) – Gambian sun squirrel

No mid-ventral white stripe, dull light grey coat, GLS <51mm. Typically in savannah.

Table 3: Data obtained from Monadgem et al. 2015

	Mean (mm)	Range (mm)
Mass	245.6	157-325
HB	205.5	188-230
Tail	248	200-305
HF	50.2	45-54
Ear	14.8	10-18
GLS	47.4	42.5-51.6

<heliosciurus gambianus image>

Heliosciurus punctatus (Temminck, 1853) – Small sun squirrel

No mid-ventral white stripe, dull dark grey-brown coat, GLS <51mm. Typically in forest.
<heliosciurus punctatus image>

Table 4: Data obtained from Monadgem et al. 2015

	Mean (mm)	Range (mm)
Mass	145.5	119-163
HB	186.7	155-206
Tail	227.3	202-270
HF	46.7	44-51
Ear	15.2	13-17
GLS	45	41.3-48

Heliosciurus rufobrachium (Waterhouse, 1842) – Red-legged sun squirrel

Red or reddish-brown on limbs, contrasting with duller dorsal coat
<heliosciurus rufobrachium image>

Table 5: Data obtained from Monadgem et al. 2015

	Mean (mm)	Range (mm)
Mass	329.3	250-388
HB	227.7	193-256
Tail	267.3	203-320
HF	53.9	47-60
Ear	15.7	10-19
GLS	52.1	46.3-56.8

Paraxerus poensis (A.Smith, 1830) – Green bush squirrel

Slender body, long legs and long tail. Dull olive green coat. Small rounded ears. 3 pairs of nipples. Small GLS <40, HF <36. No stripe, dull coat, no orange or red colouration.
<paraxerus poensis image>

Table 6: Data obtained from Monadgem et al. 2015

	Mean (mm)	Range (mm)

Mass	171.3	114-346
HB	157.9	132-179
Tail	184.6	106-218
HF	40	32-40
Ear	12.8	10-17
GLS	40.5	34.7-43.5

Protoxerus aubinnii (Gray, 1873) – Slender-tailed squirrel

Large squirrel 400-500g, uniform dark brown coat with long slender tail. Long ears. Non-bushy tail can be 1.25 times HB length. Four pairs of nipples.

<protoxerus aubinnii image>

Table 7: Data obtained from Monadgem et al. 2015

	Mean (mm)	Range (mm)
Mass	470.7	429-498
HB	253	240-267
Tail	295	273-310
HF	54.5	52-57
Ear	20.1	19-21
GLS	59.2	58.5-60.1

Protoxerus stangeri (Waterhouse, 1842) – Forest giant squirrel

550-750g, slender body with relatively short legs. Large head and long tail. Hairs of the head are white tipped. Tail is slightly longer than the body with alternating black and white (narrow) bands. 4 pairs of nipples.

<protoxerus stangeri image>

Table 8: Data obtained from Monadgem et al. 2015

	Mean (mm)	Range (mm)
Mass	616.8	393-716
HB	267.7	210-310
Tail	316.8	230-394
HF	64.9	60-72
Ear	21.4	15-25
GLS	66.4	62-70.5

Dormice

Small, resembles squirrel shaped body.

Graphiurus crassicaudatus (Jentink, 1888) – Jentink's dormouse

Larger than GLS >24mm but < 32mm, upper incisors pro-odont. Nasals parallel-sided; dorsal coat rufous or with rufous hue. Rufous coat, pro-odont upper incisors. Parallel-sided nasals with strong supraorbital ridges.

<graphiurus crassicuadatus image>

Table 9: Data obtained from Monadgem et al. 2015

	Mean (mm)	Range (mm)
Mass	23.6	19-29
HB	88.9	77-100
Tail	58.4	53-70
HF	16.4	13-19
Ear	12	7-15
GLS	25.6	24.1-27.8

Graphiurus lorraineus (Dollman, 1910) – Lorrain dormouse

Dorsal and ventral coat not clearly demarcated. Smaller than GLS <25mm, nasals broaden out distally. Coat brown or rufous. Upper incisors orthodont or opisthodont.

<graphiurus lorraineaus image>Y

Table 10: Data obtained from Monadgem et al. 2015

	Mean (mm)	Range (mm)
Mass	16.8	12-24
HB	80.5	70-93
Tail	66.8	45-77
HF	16.5	13-19
Ear	12.4	9-15
GLS	24.5	22.7-26.2

Graphiurus nagtglasii (Jentink, 1888) – Nagtglas's African dormouse

GLS >32mm, facial mask inconspicuous or absent, tail not white tipped.

<graphiurus nagtglassi image>

Table 10: Data obtained from Monadgem et al. 2015

	Mean (mm)	Range (mm)
Mass	70.2	62-77
HB	132.4	108-160
Tail	126	102-160
HF	27.3	24-31
Ear	19.4	16-22
GLS	36.3	32.9-39.9

Cricetomys

Very large rodents (HB 320-350). Long two coloured tail, small feet and large ears. Large skull GLS 65-75mm. Nocturnal rodent, omnivorous. Difficult to distinguish these species morphologically so distribution is used. C. emini is the only one present in Guinea and Sierra Leone but also consists of 4 sub-species.

Cricetomys emini (Wroughton, 1910) – Emin's pouched rat

<cricetomys emini image>

Table 11: Data obtained from Monadgem et al. 2015

	Mean (mm)	Range (mm)
Mass	1000	-
HB	328.5	300-355
Tail	381.6	420-429
HF	67.7	64-71
Ear	38.1	33-45
GLS	70.3	59.5-77.2

Lophuromys

Small to large HB 90-180, relatively short tail 50-85% of HB. Terrestrial and nocturnal genus. Omnivorous

Lophuromys sikapusi (Temminck, 1853) – Rusty-bellied brush-furred rat

Only species of the genus in West Africa west of the Sanaga River. Likely contains several cryptic species.

<lophuromys sikapusi image>

Table 12: Data obtained from Monadgem et al. 2015

	Mean (mm)	Range (mm)
Mass	63.2	34-87
HB	129.9	110-145
Tail	69	58-75
HF	23	20-25
Ear	16.2	14-18
GLS	31.1	27.6-33.5

Uranomys

Small genus HB ~105mm, short tail relative to body. Small skull. Terrestrial, nocturnal, live in deep burrows. Partly insectivorous but also feeds on cultivated crops.

Uranomys ruddi (Dollman, 1909) – Rudd's mouse

<uranomys ruddi image>

Table 13: Data obtained from Monadgem et al. 2015

	Mean (mm)	Range (mm)
Mass	33.1	26-43
HB	106.4	95-119
Tail	68.6	60-73
HF	17.1	16-19
Ear	13.4	10-15

GLS	26.4	25-27.7

Gerbillinae (subfamily)

Two species of the genus gerbilliscus are identified in SL. Relatively large 90-130g, HB 130-169mm and heavily built. Hindfoot soles are naked. Nocturnal and terrestrial. Diet is omnivorous and opportunistic.

Gerbilliscus guineae (Thomas, 1910) – Guinean gerbil

Smaller size GLS 35mm, tail 90-100% of HB with a pencil, brown tail, paler below. 2n = 50. Difficult to distinguish from co-occuring species in W. Africa without chromosomal/molecular diagnosis.
<gerbilliscus guineae image>

Table 13: Data obtained from Monadgem et al. 2015

	Mean (mm)	Range (mm)
Mass	72.8	50-136
HB	140.9	102-178
Tail	173.5	151-198
HF	33	28-38
Ear	20.7	18-24
GLS	35	33-37.5

Gerbilliscus kempi (Wroughton, 1906) – Kemp's gerbil

Longer ear 20mm. Longer tail 110-120% of HB. 2n = 36.
<gerbilliscus kempi image>

Table 14: Data obtained from Monadgem et al. 2015

	Mean (mm)	Range (mm)
Mass	97.3	70-128
HB	154.1	125-184
Tail	152.7	115-188
HF	35.5	32-41
Ear	19.6	17-23
GLS	40.1	36.3-43.3

Arvicanthis – Grass rats

Medium to large-sized rodents (35-130g, HB 100-160mm), sparsely haired, coarse scaled tail, shorter than head and body length (65-95% of HB). 6 nipples. Diurnal rodents, specialised herbivores.

Arvicanthis niloticus (É.Geoffroy, 1803) – African grass rat

No morphological species that can be used to confidently distinguish this species. Possible species complex.
<arvicanthis niloticus image>

Table 14: Data obtained from Monadgem et al. 2015

	Mean (mm)	Range (mm)
Mass	114.8	89-160

HB	156.6	127-188
Tail	129.6	92-155
HF	33	30-36
Ear	18.3	13-22
GLS	34.9	29.8-40.2

Arvicanthis rufinus (Temminck, 1853) – Guinean grass rat

Previously described as a synonym of A. niloticus.

<arvicanthis rufinus image>

Table 15: Data obtained from Monadgem et al. 2015

	Mean (mm)	Range (mm)
Mass	103.3	59-168
HB	143.8	115-177
Tail	138.2	127-146
HF	32.7	29-38
Ear	19.8	16-24
GLS	37.9	34.7-40.5

Dasymys

Medium large rodents (mass 80-120g, HB 130-170mm). Characterised by a flat, disc like face with small eyes, shaggy soft, long haired coat. Tail is shorter in length than head and body 73-95% of HB. Herbivorous and good swimmers. Prefer marshy habitats.

Dasymys rufulus (Miller, 1900) – West African shaggy rat

Only Dasymys species in W. Africa.

<dasymys rufulus image>

Table 16: Data obtained from Monadgem et al. 2015

	Mean (mm)	Range (mm)
HB	141	122-159
Tail	139	131-152
HF	28.5	25-31
Ear	19	15-23
GLS	34.4	32.5-37.2

Dephomys

Medium sized rodent, reddish brown coat. Long and sleek fur with sparse black tipped guard hairs. Ventral hairs, white tipped with grey base. Tail is 160% of HB, black in colour with rings of small scales and bristle.

Dephomys defua (Miller, 1900) – Defua rat

2n 54. Distinguished from D. eburneae by the presence of t3 on upper molar or chromosomal grounds.

<dephomys defua image>

Table 17: Data obtained from Monadgem et al. 2015

	Mean (mm)	Range (mm)

Mass	62.5	57-68
HB	124.8	112-136
Tail	191	184-195
HF	26.3	25-27
Ear	17.9	16-20
GLS	32.5	28.4-35

Grammomys

Small to medium sized rats HB 85-140mm, long tail 120-163% of HB, inconspicuous but visible tuft. Relatively small feet with 5 digits. Skull is small. Genus is arboral and nocturnal. Frugivorous or vegetarian.

Grammomys buntingi (Thomas, 1911) – Bunting's thicket rat

May overlap with G. macmillani in Ivory Coast.

Table 18: Data obtained from Monadgem et al. 2015

	Mean (mm)	Range (mm)
HB	114.4	100-125
Tail	170	165-180
HF	25	24-26
Ear	18.4	16-20
GLS	30	28.6-31.4

Hybomys

Forest mice, medium sized (Mass 50-65g, HB 110-165mm) sleek and soft coat. 1-3 black dorsal stripes. Tail is black and appears naked. Digits 1 and 5 highly reduced compared to most murines. Associated with primary and secondary tropical rainforest. Terrestrial, primarily nocturnal. Insectivorous diets.

Hybomys planifrons (Miller, 1900) – Miller's striped mouse

Narrow and tapering rostrum, 2 pairs of nipples. Cranial profile flat anteriorly. Single mid-dorsal stripe extending from the head to the base of the tail. Co-occurs with trivirgatus. 2n = 35-39.

Table 19: Data obtained from Monadgem et al. 2015

	Mean (mm)	Range (mm)
Mass	51	46-60
HB	124	120-130
Tail	97	91-104
HF	30.6	28-32
Ear	17.4	16-19
GLS	32.8	32-33.8

Hybomys trivirgatus (Temminck, 1853) – Temminck's striped mouse

Narrow and tapering rostrum, 2 pairs of nipples. Cranial profile flat anteriorly. Three mid-dorsal stripes present. 2 lateral stripes shorter and sometimes inconspicuous. 2n = 40-43.

<hypomys trivirgatus image>

Table 20: Data obtained from Monadgem et al. 2015

	Mean (mm)	Range (mm)
Mass	49.6	29-63
HB	118.7	105-130
Tail	97	85-110
HF	30.6	27-34
Ear	16.3	15-18
GLS	33.4	28.1-36.1

Hylomyscus

Small to very small (mass 10-35g, HB 70-120mm). Soft furred arboreal mice lacking guard hairs. Tail typically 120-165% of HB. Widespread in lowland and montane tropical rainforest. Arboreal, nocturnal and omnivorous.

Hylomyscus baeri (Heim de Balsac & Aellen, 1965) – Baer's wood mouse

Supraorbital ridges present, ventral coat pure white, tip of tail without obvious hairs. Four pairs of nipples.

Table 21: Data obtained from Monadgem et al. 2015

	Mean (mm)	Range (mm)
Mass	21.8	21-23
HB	105.9	98-115
Tail	128.1	111-141
HF	21.8	21-23
Ear	16.3	15-19
GLS	25.8	24.2-26.9

Hylomyscus simus (G.M.Allen & Coolidge, 1930)

Supraorbital ridges absent, incisors opisthodont (pointing backwards) GLS > 22m, incisors orthodont or slightly pro-odont. Can only be identified with certainty on molecular grounds.

Typically found in rainforest.

Table 22: Data obtained from Monadgem et al. 2015

	Mean (mm)	Range (mm)
Mass	18.5	12-22
HB	89	66-128
Tail	123	52-143
HF	19	16-20
Ear	16	13-18
GLS	23.8	20.7-25.3

Lemniscomys

Medium sized rodents (mass 40-80g, HB 90-140mm), short, tawny-furred ears. Short haired, sleek coat which has three characteristic dorsal colour patterns. The tail is white ventrally, marked with sparse black hairs and small concentric scales dorsally and may be similar or

longer than the HB length 100-125%. Medium sized skull, 25-35mm, long and narrow. Typically occur in savannas and forest clearings. Terrestrial and crepuscular. Diet varies seasonally.

Lemniscomys bellieri (Van der Straeten, 1975) – Bellier's striped grass mouse

Dorsal coat with multiple lines broken into spots. Midline flanked by 8 rows of spots not arranged into neat lines and/or spots in lower rows tending to connect to form lines; GLS <29mm. Occurs in grasslands, particularly moist savanna, avoiding forest and forest edge habitats.

<lemniscromys bellieri image>

Table 23: Data obtained from Monadgem et al. 2015

	Mean (mm)	Range (mm)
HB	109	91-127
Tail	112	94-134
HF	25.4	23-27
Ear	15.9	13-19
GLS	28.2	24.4-31.3

Lemniscomys striatus (Linnaeus, 1758) – Typical striped grass mouse

Dorsal coat with multiple lines broken into spots. Midline flanked by 8 rows of spots. GLS >29mm.

<lemniscomys striatus image>

Table 24: Data obtained from Monadgem et al. 2015

	Mean (mm)	Range (mm)
Mass	41.1	28-67
HB	115.6	97-139
Tail	124.4	96-153
HF	26.7	22-29
Ear	15.9	12-21
GLS	30.7	27.6-33.1

Lemniscomys linulus (Thomas, 1910)

Dorsal coat has a single mid-dorsal stripe. Found in drier savanna habitats, likely not in SL.

Table 25: Data obtained from Monadgem et al. 2015

	Mean (mm)	Range (mm)
Mass	38.7	21-23
HB	105.4	98-115
Tail	111.4	111-141
HF	26	21-23
Ear	16.6	15-19
GLS	27.3	24.2-26.9

Malacomys

Large (mass 60-80g. HB 130-160mm), large naked ears, short, soft, fine fur, lacking guard hairs. Tail is bicoloured, relatively long 130-136% of HB. Long slender pale-coloured hindfoot, >31mm 25% of HB. Terrestrial, nocturnal and feed on slugs, earthworms and plant parts.

Malacomys edwardsi (Rochebrune, 1885) – Edward's swamp rat

HF <35mm 2 pairs of nipples.

<malacomys edwardsi image>

Table 26: Data obtained from Monadgem et al. 2015

	Mean (mm)	Range (mm)
Mass	64.5	46-80
HB	140.1	126-160
Tail	168.8	155-180
HF	34.2	31-37
Ear	25.7	24-28
GLS	37.9	34.9-39.6

Mastomys

Females of these rodents are recognisable by having 16-24 pairs of mammae. Wide distribution in savannas, grasslands and agricultural habitats. Terrestrial and nocturnal, granivorous and omnivorous.

Mastomys erythroleucus (Temminck, 1853) – Guinea multimammate mouse

Can only be distinguished with certainty from other species of Mastomys on chromosomal or molecular grounds. 2n = 38, FN = 50-60.

<mastomys erythroleucus image>

Table 27: Data obtained from Monadgem et al. 2015

	Mean (mm)	Range (mm)
Mass	51.3	32-107
HB	129.9	92-178
Tail	115.8	81-151
HF	23.4	20-27
Ear	18.8	15-23
GLS	28.8	23.7-34.3

Mastomys natalensis (A.Smith, 1834) – Natal multimammate mouse

Can only be distinguished with certainty from other mastomys species on chromosomal and molecular grounds. 9-12 equally spaced pairs of nipples are present in this species. 2n = 32, FN = 52-53. Typically associated with agricultural fields and homes, but also in savannas and grasslands, noted population abundance surges after disturbance such as fire.

<mastomys natalensis image>

Table 28: Data obtained from Monadgem et al. 2015

	Mean (mm)	Range (mm)
Mass	40.8	15-98
HB	108.1	76-155
Tail	102.1	70-174

HF	22.9	18-26
Ear	17.3	12-22
GLS	28.3	22.3-34.8

Mus

Easily identified by their very small to small body size (2-18g, HB 37-97mm for nannomys, 13-30g, HB 72-92mm in mus). They have naked tail. Their dorsal coats are comprised of short sleek fur.

Mus baoulei (Vermeiren & Verheyen, 1980) – Baolule's mouse

Red markings close to the ear openings. Short tail 50-65% of HB. 2n = 2-/21. Typically found in savanna habitats.

Table 29: Data obtained from Monadgem et al. 2015

	Mean (mm)	Range (mm)
Mass	6.8	6-8
HB	65.9	56-70
Tail	42.6	38-45
HF	13.1	12-15
Ear	10.6	10-12
GLS	18.7	18-20.3

Mus mattheyi (F.Petter, 1969)

2n = 36, associated with savanna habitats.

<mus mattheyi image>

Table 30: Data obtained from Monadgem et al. 2015

	Mean (mm)	Range (mm)
Mass	6.5	6-7
HB	52.9	45-61
Tail	37.6	32-41
HF	12.1	11-14
Ear	8.8	7-10
GLS	17.4	15.3-19.7

Mus minutoides (A.Smith, 1834) – African pygmy mouse

Short tail 75% of HB, pure white belly, brownish-buff to brownish orange coat. 2n = 18-36. Typically savanna and grassland habitats. GLS <20mm, upper incisors orthodont or opisthodont.

<mus minutoides image>

Table 31: Data obtained from Monadgem et al. 2015

	Mean (mm)	Range (mm)
Mass	6.8	3-12
HB	56.5	40-72
Tail	42.7	27-57
HF	13.1	9-16
Ear	9.3	6-12
GLS	18.2	17-19.2

Mus musculoides (Temminck, 1853)

Cannot be diagnosed morphologically from minutoides. 2n 18-19.
<mus musculoides image>

Table 32: Data obtained from Monadgem et al. 2015

	Mean (mm)	Range (mm)
Mass	6.8	6-9
HB	63.8	58-75
Tail	49.4	47-53
HF	13	12-14
Ear	8.5	7-10
GLS	17.3	16.7-18.1

Mus musculus (Linnaeus, 1758)

Only member of subgenus mus in SSA. Typically commensal species. No stripes, upper incisors have posterior notch, upper 3rd molar has two laminae.
<mus musculus image>

Table 33: Data obtained from Monadgem et al. 2015

	Mean (mm)	Range (mm)
Mass	14.5	10-20
HB	77.4	61-109
Tail	78.6	62-88
HF	17.3	15-20
Ear	12.7	12-15
GLS	20.5	18.2-21.8

Mus setulosus (Peters, 1876) – Peters's mouse

White belly, small, GLS <23mm>20mm, upper incisors orthodont or opisthodont, dorsal coat without stripes. Largest of the subgenus Nannomys. Dorsal coat is dull blackish-brown, not as bright as in minutoides or musculoides. Possibly a species complex.

Table 34: Data obtained from Monadgem et al. 2015

	Mean (mm)	Range (mm)
Mass	13.7	8-23
HB	72.3	52-84
Tail	53.4	43-63
HF	15.4	13-17
Ear	11	8-14
GLS	20.9	19.9-22.4

Oenomys

Medium to large-sized rats, 90-110g, HB 140-160mm. Have a sparsely haired tail, bicoloured tail, relatively long 110% HB. Small, densely furred ears. Coat is soft with long guard hairs and typically greyish brown to bright reddish, prominently darker orange around the nose, rump and anal area. Occur in grassy clearings within or at the edge of rainforests. Terrestrial, both diurnal and nocturnal. Diet is herbivorous.

Oenomys ornatus (Thomas, 1911) – Ghana rufous-nosed rat
Tail 130% of HB, 2n = 46.

Limited morphological data

Praomys
Small to medium sized mice 25-60g, HB 100-130mm, soft coat which is greyish buffy to almost black to reddish brown dorsally and greyish to white ventrally. Ears are large and rounded. The tail is finely scaled, appears naked and is longer than the HB 110-130%. Fewer nipples than the similarly sized mastomys. Mostly associated with forest and woodland, more numerous in disturbed forests. Nocturnal terrestrial rodents.

Praomys daltoni (Thomas, 1892)
7 palatal ridges, shorter hindfoot. Pure white ventral coat which is pure white (hairs with white bases).

Table 35: Data obtained from Monadgem et al. 2015

	Mean (mm)	Range (mm)
Mass	41	24-66
HB	112.6	98-133
Tail	125.1	107-142
HF	22.1	20-25
Ear	17.6	16-20
GLS	28.6	25.8-32.2

Praomys rostratus (Miller, 1900)
Can be distinguished from tullbergi on molecular grounds, but is also slightly larger with significant overlap. 2n = 34, FN = 32.

Table 36: Data obtained from Monadgem et al. 2015

	Mean (mm)	Range (mm)
Mass	50.5	31-73
HB	129.5	109-147
Tail	145.1	124-171
HF	26.4	24-31
Ear	18.9	17-21
GLS	34	30-36.9

Praomys tullbergi (Thomas, 1894)
9 palatal ridges.

Table 37: Data obtained from Monadgem et al. 2015

	Mean (mm)	Range (mm)
Mass	37.9	28-54
HB	119.3	108-131
Tail	139.5	115-153
HF	24.1	22-26
Ear	18.7	17-21
GLS	31.5	29.1-34.3

Rattus

Large to very large (100-200g, HB 160-210mm). Characteristic coarse blackish-brown to brown coat. Multiple guard hairs and ventral coat which is usually grey or white. Long and thin tail 115-120% of HB. HF >30mm. Large and transluscent ears. 5-6 pairs of nipples. Nocturnal and terrestrial, predominantly commensal.

Rattus rattus (Linnaeus, 1758)

GLS <45mm, tail >100% of HB, ears longer ~22mm

<rattus rattus image>

Table 38: Data obtained from Monadgem et al. 2015

	Mean (mm)	Range (mm)
Mass	115.2	56-257
HB	168.3	100-342
Tail	178.9	95-250
HF	33.8	23-42
Ear	21.1	16-31
GLS	39.9	34.9-44.7

	Mean	Min	Max
Mass	115.2	56	257
HB	168.3	100	342
Tail	178.9	95	250
HF	33.8	23	42
Ear	21.2	16	31
GLS	39.9	34.9	44.7

Crocidura

Crocidura buettikoferi (Jentink, 1888) – Buettikofer's shrew

Medium sized, dark coloured shrew. Dorsal coat dark chocolate brown. Ventral coat paler, greyinsh brown. Dark limbs. Tail 70% of HB, dark, sparsely covered with hairs and bristles. Typically found in grassland habitats, forest relicts in derived savanna, secondary growth, cocoa plantations and cleared land. Feeds on insects.

Table 39: Data obtained from Kingdon and Happold 2013

HB	72-100mm	Weight	
Tail	51-62mm	CI	21.4-22.7mm
HF	12-15mm	GLS	
Ear	7-9.5mm	GWS	9.2-9.9mm

Crocidura crossei (Thomas, 1895) – Crosse's shrew

2n = 44, FN = 66. Small shrew. Dorsal coat slaty-grey or greyish brown to chocolate brown. Ventral coat greyish and paler. Chin white. Limbs usually dark. Tail relatively long >84% of HB, quite stout, grey-brown above and pale below. Slender rostrum. Rainforest individuals are generally darker than those in forest-savanna habitats. Forest habitats, including lowland rainforest, relic forests in savanna.

Table 40: Data obtained from Kingdon and Happold 2013

HB	54-80mm	Weight	6.3-8g
Tail	48-57mm	CI	18.5-19.9mm
HF	10-12mm	GLS	
Ear	6-8mm	GWS	7.8-8.5

Crocidura denti (Dollman, 1915) – Dent's shrew

Perhaps includes multiple species. Small dark brown shrew with hairy tail. Dorsal coat dark brown, ventral coat slate grey. Tail relatively long, 65-70% of HB, brown above, pale below, hair on 66-75%.

Table 41: Data obtained from Kingdon and Happold 2013

HB	63mm	Weight	10.5g (8-12)
Tail	46mm	CI	20.6mm
HF	13mm	GLS	
Ear	8mm	GWS	9.3mm

Crocidura fuscomurina – Bicolored musk shrew

Very small, predominantly grey-brown shrew grizzled with grey. Dorsal coat grey-brown, hairs slaty-grey at base with a fawn subterminal band and brown or buffy-brown tip. Ventral coat grey, with sometimes yellow tinge. Dorsal and ventral colours clearly delineated. Fore and hindfeet pale brown to off-white. Tail ~65% of HB, pale to dark brown above, paler below. Found in woodland savannas and semi-arid regions.
<crocidura fuscomurina image>

Table 42: Data obtained from Kingdon and Happold 2013

HB	57.9 (48-75) mm	Weight	3.7g (2-5)
Tail	38.5 (27.5-48)mm	CI	16.2mm (15.1-17.4)
HF	9.8 (8-11)mm	GLS	
Ear	7.6 (6.5-9)mm	GWS	7.2mm (6.7-7.7)

Crocidura lamottei (Heuglin, 1865) – Lamotte's shrew

Medium sized shrew with relatively pale coloured coat. Dorsal coat pale brown to grey-brown, without flecking. Ventral coat grey or silvery grey washed with yellow tint, hairs medium grey at base, creamy-grey at tip. Ears conspicuous and naked, not concealed by coat. Hindfoot relatively short. Fore and hindlimbs flesh coloured, covered with short pale brown hairs. Medium length tail (53% of HB), thick, broad at base narrowing towards tip, flesh coloured with reddish blotches. Found primarily in dry forest, grasslands in savanna, rocky hillside and grassy clearings.

Table 43: Data obtained from Kingdon and Happold 2013

HB	85-90 mm	Weight	18-23g
Tail	35.5-55 mm	CI	24.7-26mm
HF	14-16 mm	GLS	
Ear	11-12 mm	GWS	10.1-10.6mm

Crocidura muricauda (Miller, 1900) – West African long-tailed shrew

Small shrew, large ears and a long tail. Soft coat. Dorsal coat greyish brown, hairs grey at base, brown at tip. Ventral coat white-grey, hairs grey at base, white at tip. Ears large in relation to head, more or less naked. Fore and hindfeet flesh-coloured, minimally covered with short white hairs. Tail pale brown, extremely long (120-150% of HB), not bicoloured, tail hairs tend to be short and less visible. Skull long and narrow. Found in rainforest and secondary forest.

Table 44: Data obtained from Kingdon and Happold 2013

HB	61 (56-66) mm	Weight	4.4 (3-8) g
Tail	77 (62-95) mm	CI	18.2 (17.8-18.8) mm
HF	12.2 (11.8-13) mm	GLS	
Ear	9 (8.5-10) mm	GWS	8.4 mm

Crocidura nimbae (Heim de Balsac, 1956) – Nimba shrew

Medium-sized dark shrew with relatively short tail. Coat short and dense with silky sheen, hairs 3mm. Dorsal coat, dark brownish-grey to grey with sparse flecking, hairs dark grey at base becoming browner towards the tip. Ventral coat slightly paler and greyer and greyer than dorsal coat. Ears conspicuous, not concealed by coat. Flank gland visible as small oval patch of bare skin. Fore and hindfeet flesh coloured with short brown hairs on the upper surface, whitish hairs on the toes. Tail relatively short 40% of HB, bicoloured, dark brownish grey above, paler ventrally. Habitat, submontane and lowland rainforest.

Table 45: Data obtained from Kingdon and Happold 2013

HB	81.7 (75-90) mm	Weight	12.5 (8-19) g
Tail	53 (50-70) mm	CI	26 (25.5-26.5) mm
HF	16.5 (16-17) mm	GLS	
Ear		GWS	10 mm

Crocidura obscurior (Heim de Balsac, 1958) – West African pygmy shrew

Minute to very small dark brown shrew. Soft coat, dense and velvety. Dorsal coat dark brown with slight russet tinge and silky sheen, hairs almost unicoloured, some with a dark brown tip. Ventral coat greyish brown. Head similar to doral coat. Ears darkly pigmented and not covered by coat. Chin and throat similar to ventral coat. Tail 60-70% of HB, dark brown above and paler below, long white hairs on tail.

Table 46: Data obtained from Kingdon and Happold 2013

HB	45-50 mm	Weight	2.5-4 g
Tail	30-35 mm	CI	15.6-15.9 mm
HF	10 mm	GLS	
Ear	6-8 mm	GWS	7.1-7.3 mm

Crocidura olivieri (Lesson, 1827) – African giant shrew

Very large shrew, variable in colour. Dorsal coat, reddish brown to dark brown or black. Ventral coat buffy brown to dark grey. Tail relatively long (70-80% of HB). Thick with many short bristles. 6 nipples. Nocturnal, with peaks of activity around dawn.
<crocidura olivieri image>

Table 47: Data obtained from Kingdon and Happold 2013

HB	110-140 mm	Weight	36-65 g
Tail	85-100 mm	CI	32-34.3 mm
HF	21-23 mm	GLS	
Ear	12-14 mm	GWS	13.2-14.4 mm

Crocidura poensis (Fraser, 1843) – Fraser's musk shrew
Medium sized mush shrew. Dorsal coat dark brown to black. Ventral coat paler, greyish brown. Ear prominent. Limbs dark. Tail relatively long (75% of HB), thin and dark, covered with short bristles.

Table 48: Data obtained from Kingdon and Happold 2013

HB	75-98 mm	Weight	9-17 g
Tail	48-64 mm	CI	23.7-24.5 mm
HF	15-18 mm	GLS	
Ear	9-11 mm	GWS	10.1-10.5 mm

Crocidura theresae (Heim de Balsac, 1968) – Therese's shrew
Medium to large shrew. Dorsal coat grey to brown. Ventral coat paler. Fore and hindfeet brown. Tail medium to long (39-62% of HB), thickset with sparse brown hairs.

Table 49: Data obtained from Kingdon and Happold 2013

HB	91.3 (78-101) mm	Weight	13.3 (7-18.4) g
Tail	47.5 (34-55) mm	CI	22.8 (21.3-24.4) mm
HF	14.8 (13-16) mm	GLS	
Ear		GWS	9.6 (8.8-10.2) mm

Suncus

Suncus megalura (Jentink, 1888) – Climbing dwarf shrew
Small shrew, with an extremely long, thin tail and long hindfeet. Coat soft and moderately dense. Dorsal coat greyish with brown tinge, hairs grey on basal two third, brown on terminal third. Ventral coat off-white to pale. Head slender with long, narrow, pointed muzzle, small eyes and rounded ears. Fore and hindfeet brown, slender and relatively long (25% of HB). Tail extremely long (118% of HB). 6 nipples.
<suncus megalura image>

Table 50: Data obtained from Kingdon and Happold 2013

HB	60.8 (50-65) mm	Weight	5.5 (3-8) g
Tail	88.8 (82-97) mm	CI	18 (16.8-18.6) mm
HF	15.5 (14-17) mm	GLS	
Ear	8.3 (6-10) mm	GWS	8.2 (7.8-8.5) mm
